# A method for AI assisted human interpretation of neonatal EEG

**DOI:** 10.1038/s41598-022-14894-4

**Published:** 2022-06-29

**Authors:** Sergi Gomez-Quintana, Alison O’Shea, Andreea Factor, Emanuel Popovici, Andriy Temko

**Affiliations:** 1 Electrical and Electronic Engineering, University College Cork, Cork, Ireland; 2 Department of Computer Science, Munster Technological University, Cork, Ireland; 3 Department of Anatomy and Neuroscience, University College Cork, Cork, Ireland

**Keywords:** Neonatal brain damage, Epilepsy, Biomedical engineering

## Abstract

The study proposes a novel method to empower healthcare professionals to interact and leverage AI decision support in an intuitive manner using auditory senses. The method’s suitability is assessed through acoustic detection of the presence of neonatal seizures in electroencephalography (EEG). Neurophysiologists use EEG recordings to identify seizures visually. However, neurophysiological expertise is expensive and not available 24/7, even in tertiary hospitals. Other neonatal and pediatric medical professionals (nurses, doctors, etc.) can make erroneous interpretations of highly complex EEG signals. While artificial intelligence (AI) has been widely used to provide objective decision support for EEG analysis, AI decisions are not always explainable. This work developed a solution to combine AI algorithms with a human-centric intuitive EEG interpretation method. Specifically, EEG is converted to sound using an AI-driven attention mechanism. The perceptual characteristics of seizure events can be heard using this method, and an hour of EEG can be analysed in five seconds. A survey that has been conducted among targeted end-users on a publicly available dataset has demonstrated that not only does it drastically reduce the burden of reviewing the EEG data, but also the obtained accuracy is on par with experienced neurophysiologists trained to interpret neonatal EEG. It is also shown that the proposed communion of a medical professional and AI outperforms AI alone by empowering the human with little or no experience to leverage AI attention mechanisms to enhance the perceptual characteristics of seizure events.

## Introduction

In 2019, 2.4 million neonatal deaths occurred globally^1^. Most neonatal deaths occur during the first week of life, and about 1 million newborns die within the first 24 h. The vast majority occurred in developing countries, with hypoxic-ischemic encephalopathy (HIE) due to birth asphyxia being one of the major causes of high child mortality^2^. While the incidence of encephalopathy ranges from 1 to 8 per 1000 live births in developed countries, it is as high as 26 per 1000 live births in underdeveloped countries^3^. Neonatal seizures are associated with various acute illnesses such as strokes, HIE or infections^4^. The reported incidence of such events varies from 1.5 to 3.5 out of 1000 newborns^5–7^. The mortality rate for those affected by seizures is approximately 10% (range 7–16%), and permanent neurological disability is estimated at around 50% of those that survive^8–11^. Most of those neonatal deaths could have been prevented with optimal care^12^.

Detection of seizures is a challenging clinical task. While seizures are often associated with clinical signs in children and adults, including involuntary jerking movements of the arms and legs, eye blinkings, or difficulty breathing, less than 10% are accompanied by documentable physical manifestations in the neonatal population. Monitoring electrical brain activity through electroencephalography (EEG) is the only way to detect seizures accurately^13,14^. While EEG has become a gold standard tool in neonatal neurophysiology and most neonatal units in the developed world have access to EEG acquisition, only some have immediate availability of neurophysiological expertise to interpret the signal^15^. In addition, EEG is a highly complex signal, even more so for neonates where the brain is still developing^16^. While multi-channel EEG monitoring can last several hours to several days, seizures are infrequent events. Therefore, a simpler representation of EEG is often used to facilitate the interpretation of long and complex EEG signals, such as amplitude-integrated EEG (aEEG). This method represents the signal as temporally smoothed and energy-compressed waveforms^17^. While aEEG allows observing several hours of EEG on a single screen page, seizures become difficult to detect visually due to limited spatial coverage, attenuation of short duration seizures due to smoothing, and false alarms are frequently caused by prolonging energy artefacts^18,19^.

Raw multi-channel EEG monitoring is required to detect all seizures. The raw EEG signal interpretation requires extensive clinical expertise and years of training. Multi-channel EEG, especially continuous multi-channel EEG obtained from long real-life clinical monitoring, is often corrupted with all sorts of artefacts, some of which can resemble waveform patterns similar to seizures. When EEG recordings last for several days, detecting relatively rare seizure events becomes a challenging and highly time-consuming process. Even among experienced EEG experts, there is still a substantial disagreement when detecting seizure events^20^. Moreover, the level of expertise of a healthcare professional required to identify seizures scales with the complexity of the data to interpret—clinical manifestations can be interpreted by nurses, aEEG interpretation requires neonatologists, whereas full EEG requires neurophysiologists. The higher the level of experience of a healthcare professional is, the smaller the chances of its availability onsite 24/7.

Artificial intelligence (AI) has become a popular tool to assist medical professionals in interpreting EEG signals. AI aims to close the gap between the availability of interpretation expertise and detection timeliness and accuracy, particularly in detecting seizure events. For years, automated seizure detection was tackled as a machine-learning (ML) problem in a well-established two-step approach consisting of (a) the summarization EEG data into a set of hand-crafted informative characteristics called features and (b) the usage of classifiers that learn the mapping between features and the labels. The works undertaking this approach differ mainly on the selected features and the choice of the classifier^21–26^. Contrary to these approaches, deep learning (DL) can directly learn the representation of the relevant information from the raw EEG data avoiding time-consuming feature engineering efforts^27–31^.

AI models are not error-free. They are often subject to adversarial attacks^32,33^. In a clinical environment, errors can result in wrong decisions concerning a diagnosis or a treatment choice, threatening and putting the overall patient’s health and well-being at risk. Therefore, it is of utmost importance to understand the nature of the errors, and the availability of an explainable framework to trace back those errors to underlying clinical causes is of utmost importance^32^. Explainable AI and saliency detection are the research areas that aim to mitigate the risks. Medical professionals require the option to understand how and why a machine decision has been made. However, there is a clear trade-off between the performance of a machine learning model and its ability to produce explainable and interpretable predictions. For example, the feature-based neonatal seizure detection approaches could explain which feature contributed most to the wrong decision^34^. However, the performance of such systems is significantly lower than that of deep learning methods^35^. The black-box models, like neural networks, tend to extract most of the information from data to beat the state-of-the-art performance without explaining and interpreting their decisions.

Alternative methods such as EEG sonification have been developed to detect neonatal seizures^36^ to assure the interpretability of clinical decisions while still simplifying the analysis process of complex EEG signals. Sonification is the process by which data are represented through sound in order to be displayed acoustically. Sonification has been proven useful in various contexts involving pattern recognition because such patterns are implicitly mapped into sound as distinguishable rhythms or tones^37^. For example, in medicine and healthcare, all sorts of biological signals are used to monitor the patient’s health, and either the presence or absence of such patterns in the data is highly informative. An example of that is the auscultation of the heart sounds using a stethoscope, a simple yet highly informative clinical examination routine used since 1838^38^.

Interestingly, acoustic perception can sometimes be even more intuitive than sight when receiving sensory information. An example of that is language itself, which is naturally self-learned by hearing during the first year of life, whereas reading (which uses the sense of sight) needs to be explicitly taught, and partly because of that, it is considered to be a less natural way to acquire information^39^. The same idea can be explored when interpreting long time-series data such as EEG. However, the task is even more complex due to multiple concurrent channels of EEG, which need to be read/analysed simultaneously.

Neonatal seizures in EEG present a specific characteristic evolving rhythmic pattern with temporal and often spatial evolution^40,41^. Morphological changes in temporal signals are often more naturally perceived auditory rather than visually. Historically, in early paper-based EEG monitoring machines, seizures could be heard as rhythmic and evolving pen movements. Most of the frequential content of neonatal EEG falls in the frequency range from 0.5 to 13 Hz approximately^42^, and the audible spectrum in humans ranges from 20 Hz to 20 kHz^43^, meaning that EEG can not be directly heard in its original time-series representation.

Earlier works in EEG sonification simply increased the sampling frequency of the EEG signal to raise its frequency content to the audible range, assuming the collateral effect of time compression^44,45^. The increased factor of the sampling frequency can be inconvenient as too large factors can make seizure events too short and therefore missed. More sophisticated methods have been developed since these earlier studies. Sonification based on tone synthesis is described in^46,47^. A musical approach where EEG amplitude is mapped into musical notes was developed^48^. Another method used EEG to modulate a voicelike synthesiser^49^. However, those approaches do not convey the totality of the EEG information but are rather partially guided by EEG. The phase vocoder (PV) was firstly used for EEG seizure detection by raising the frequency content of the EEG into the audible range without affecting the time scale^50^. In the same context of seizure detection, a subsequent study compared the PV with a novel FM/AM sonification, showing interesting results on why the perception of seizures increases with quicker playbacks^36^.

However, all the previous works in EEG sonification considered a constant time-compression factor, meaning that EEG data are compressed in time always at the same rate. Because seizures can be rare events, the constant time-compression factor results in a trade-off between the higher level of perception of short seizure events at the expense of reviewing long recordings versus having less listening overhead at the cost of missing occasional short seizure events—a shared disadvantage with aEEG^51^.

In this study, a solution for a quick preview of large multi-channel EEG recordings is presented based on the PV as in^36,50^, with an addition of an AI guided time-compression factor which serves as an attention mechanism^51^. In this manner, the accuracy of the AI method is combined with interpretability and intuitiveness of sonification to allow for quick and accurate decision making. In further subsections, we discuss the advantages and drawbacks of the AI sonification method, comparing its speed and accuracy with that of full EEG annotators and with AI alone, alongside a deep analysis of the errors made. The developed technique utilizes AI as an attention mechanism. In particular, the AI probabilities are used to modulate the speed-up factor of the EEG sonification, maintaining the focus on the EEG regions of interest (possible seizures) while allowing for a quick pass over long segments of background EEG.

The main contributions of this study are:

- A novel method of AI-driven spatial neonatal EEG sonification is presented; the AI modulates the audio playback speed to enhance the perception of seizures, which are mapped into longer duration in the acoustic domain compared to the rest of EEG data.
- An extensive survey is designed and conducted online among the targeted end-users (healthcare professionals) to assess the level of accuracy of the developed method for the detection of the presence of neonatal seizures on a publicly available dataset.
- The survey results are compared to the performance obtained by the three neonatal neurophysiologists who annotated the dataset on raw multi-channel EEG.

### AI-driven sonification algorithm

To make neonatal EEG analysis more accurate, pervasive and ubiquitous, reducing the gap between the complexity of the signal and the level of expertise needed for its analysis must be reduced while ensuring transparency in the interpretation of each decision is paramount. The method outlined in this section aims to facilitate quick and accurate diagnosis by healthcare professionals without dedicated EEG interpretation expertise while keeping the doctor at the centre of the decision-making process.

**Figure 1:**
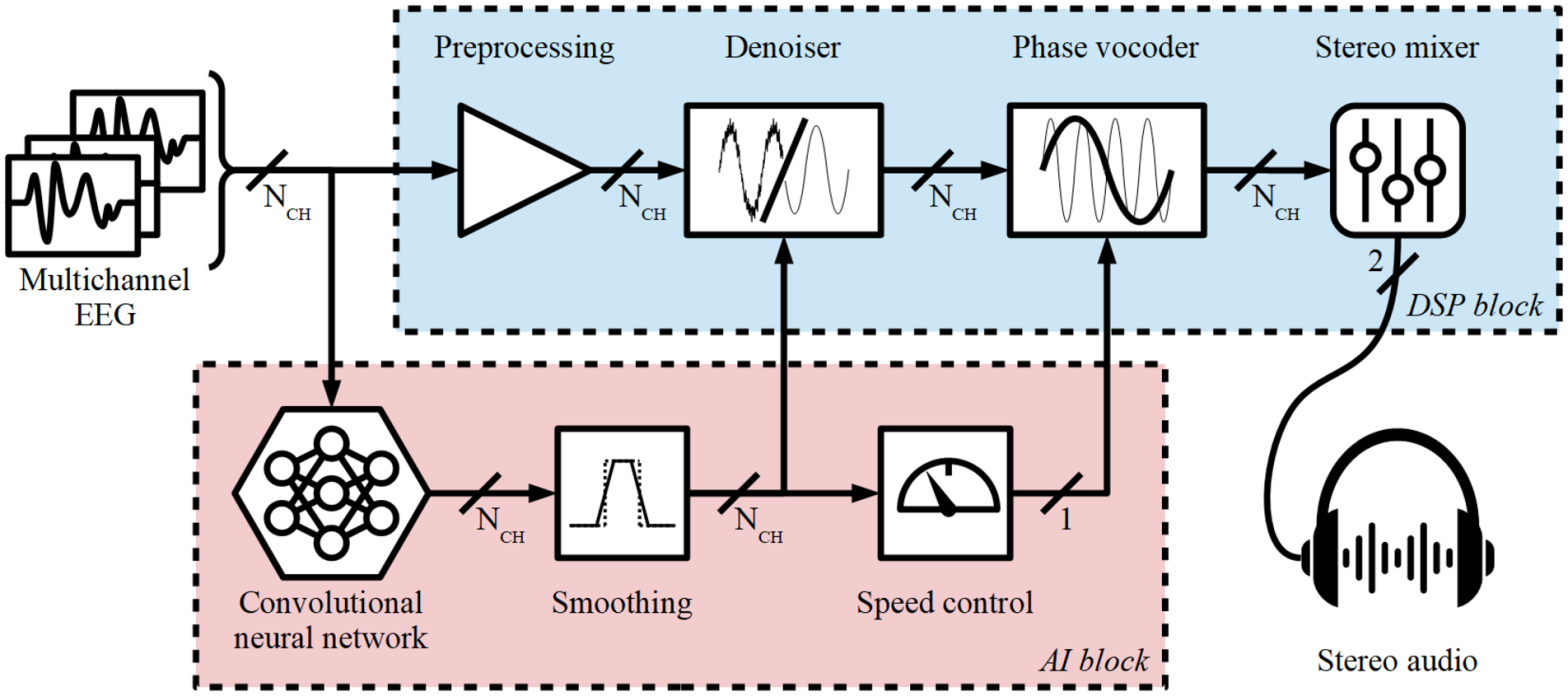
AI-driven sonification algorithm block diagram.

The outline of the proposed algorithm is shown in Fig. 1. The algorithm takes multi-channel neonatal EEG as input and delivers stereo audio that captures the content of a few hours of EEG data into a few seconds of sound. The algorithm is constituted of two fundamental blocks: the digital signal processing (DSP) block and the AI block. The DSP block converts the EEG data into audio. The EEG signals are pre-processed and denoised before the phase vocoder lifts the frequency content into an audible frequency range. While all those steps are applied in parallel to each EEG channel, the stereo mixer combines all the incoming audio tracks from each EEG channel into left–right stereo audio channels by mapping each track into a virtual 2D space to capture the relative positions of the electrodes. The AI block is used to modulate several aspects of the DSP block: it analyses the segment of EEG data, and every second provides the probability of a seizure event happening for each time segment of EEG. These probabilities control the variable time-compression rate as a function of the seizure likelihood, serving as an AI-driven attention mechanism that modulates the perceived audio accordingly.

A detailed description of each subroutine of the algorithm is available as Supplementary Methods. The following paragraphs will briefly overview the main functional load of each block.

The signal pre-processing stage applies a set of deterministic operations to adequate the EEG signals before it enters the subsequent stages of the sonification algorithm, reducing the noise level outside the band of interest and EEG artifacts^52^. A major component of this block is the attenuation of ECG artifact. The denoising block aims to emphasize any activity that deviates from the average activity in the recording by taking into account the level of seizure-ness of such activity. For this, a variant of spectral subtraction utilized in speech processing^53^ is used to reduce the amplitude of those EEG sections that do not contain seizure events.

**Figure 2:**
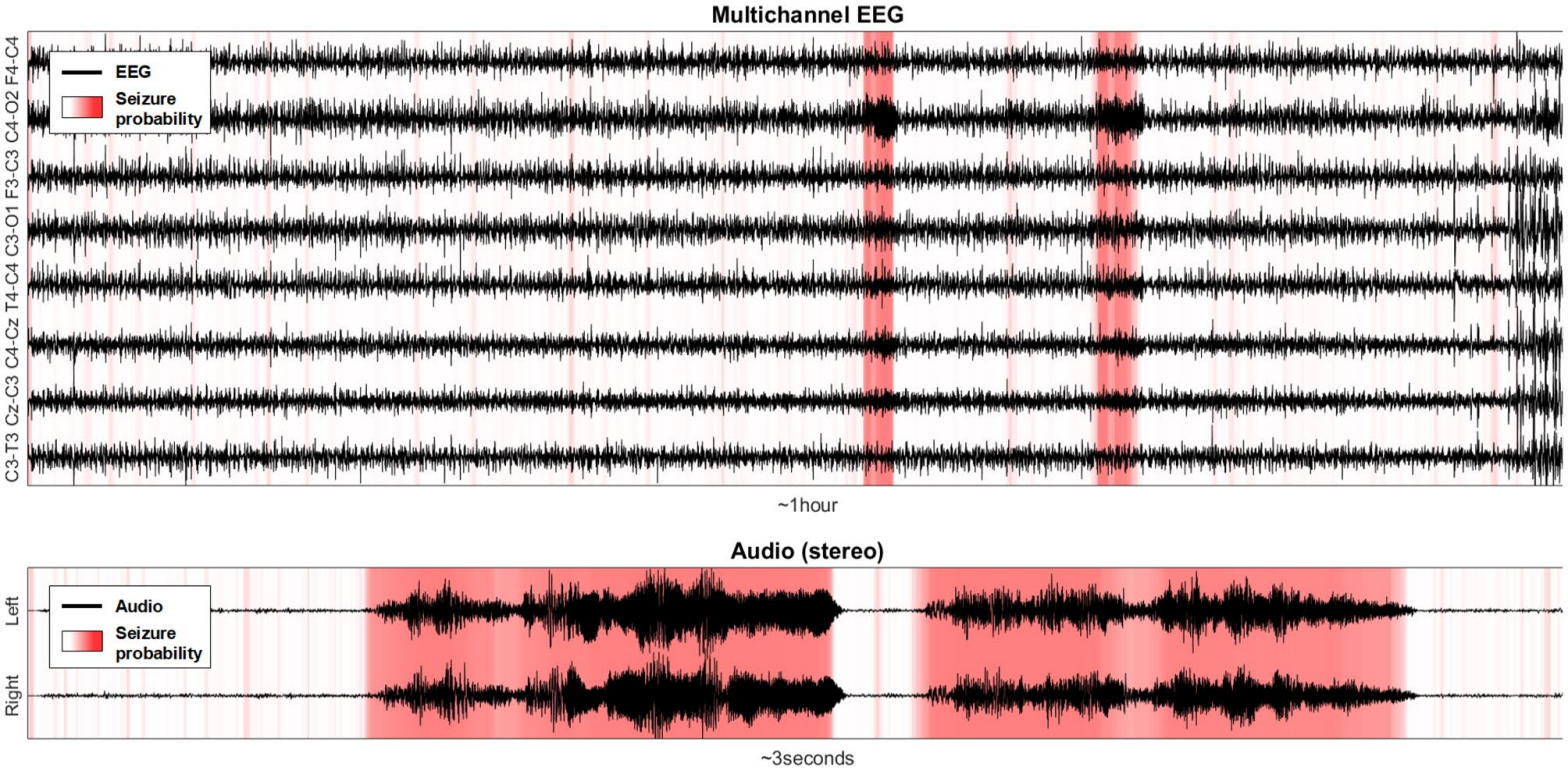
AI-driven sonification algorithm demo (EEG31 in Helsinki dataset). EEG is converted into audio and compressed non-uniformly in time as function of the seizure probability given by an AI algorithm (best seen in color).

The phase vocoder (PV) is a well-known technique to manipulate signals in the time domain by preserving their spectral properties. The PV was first developed to stretch speech signals in time to encode voice data (hence the term vocoder)^54^. Although this technique was first applied to speech, it also has been used to manipulate various types of audio^55^ and non-audio signals^56,57^. This work uses the phase vocoder to convert EEG into sound by shifting the EEG frequencies into the audible range. The new variant of the phase vocoder is derived. The new proposed algorithm allows using a time-variable stretching factor that dictates how much a particular segment of EEG gets compressed in audio. Depending on the signal properties, the stretching factor becomes dynamic and drives the EEG signal's AI interpretation. Figure 2 illustrates an example of conversion of 1 h of EEG with two seizures to 3 s of audio.

The AI block describes the AI model utilized in our work to detect seizure events. This deep learning architecture has been previously developed in^51^. The model outputs the probability of seizure for a given EEG segment and is used subsequently to inform the stretching factor. Eventually, the stereo mixer combines multiple EEG channels into stereo audio. A simple model based exclusively on the precedence effect due to the interaural time delay is used to accomplish this goal by virtually situating each audio source in a 2D plane in relation to an imaginary listener.

## Experimental design and metrics

### Dataset

The publicly available neonatal EEG dataset provided by the Helsinki University Hospital is used in this study^58^. This dataset was obtained from 79 neonates admitted to the NICU, and recordings were performed due to suspicion of seizures. It contains excerpts of 1–2 h multi-channel EEG recordings at a sampling frequency of 256 Hz stored in a referential montage with 19 electrodes positioned as per the international 10–20 standard, including a recording reference at the midline. The presence of seizures in these patients was annotated independently by three experts with over ten years of experience in visual interpretation of neonatal EEG. The annotations are represented by the timestamps for the onsets and the duration of the seizures, with no information about the specific spatial location (EEG channel) of the seizure event. All annotated seizure events were at least 10 s long. According to the annotations, by consensus regarding the presence or absence of seizures in each recording, 39 neonates had seizures (at least one seizure event detected by all experts), 22 were seizure-free by consensus, and the diagnosis of the remaining 18 patients varied among experts. In this study, the majority vote among annotators was considered the ground truth of reference, with 47 patients with seizures and 32 with no seizures annotated by at least two out of three annotators.

According to the dataset annotations, considering the consensus (agreement from all three annotators) 39 neonates had seizures, and 22 were seizure-free, while the remaining 18 patients had no consensus agreement regarding the presence/absence of seizures. Considering majority voting (minimum of two annotators agreeing), 47 neonates had seizures, and 32 were seizure-free.

### Design of the survey

A total of 79 multi-channel 1–2 h EEG recordings were sonified and converted into 79 audio samples, respectively. The level of accuracy of the developed AI-driven sonification algorithm was assessed in a survey. The survey participants were asked to distinguish the recordings with any sign of seizures from those with no sign of seizures by just listening to the audio samples. In other words, in each recording, the survey only asked about the presence/absence of seizures regardless of the actual number of seizure events or their duration.

**Figure 3:**
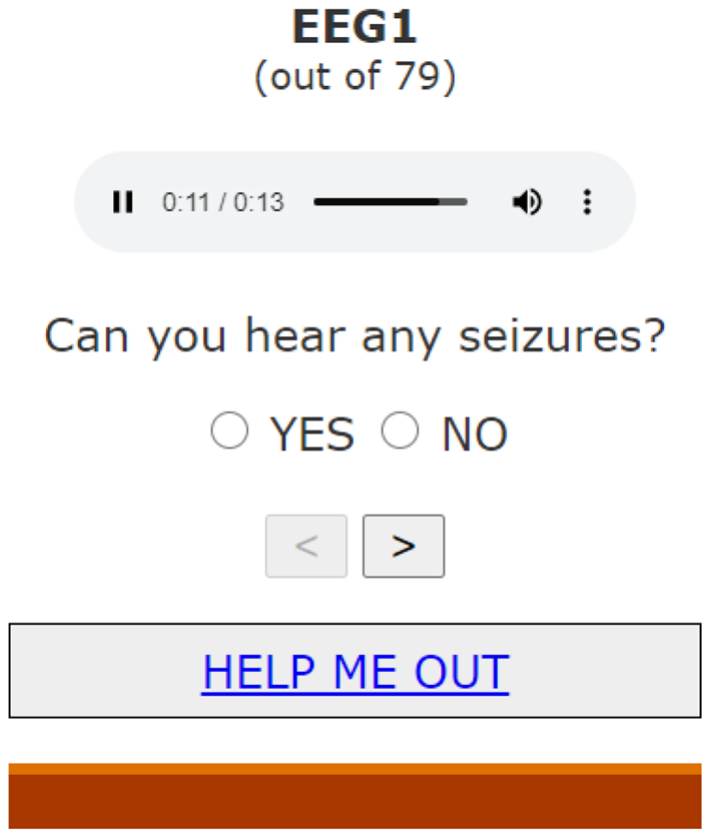
Snapshot of web survey to assess the AI-driven sonification algorithm.

An online platform was developed to conduct the survey (http://sergigomezquintana.github.io/EEGsoundSurvey). As shown in Fig. 3, a simple graphical user interface was designed to display the sonified examples and allow a participant to provide an answer and move to the next sample.

A subset of five audio samples was chosen as training examples for the participants: one containing an obvious seizure, two with less obvious seizures (short and low amplitude), one with normal background EEG and one containing ECG artefact. The examples were carefully selected to show a good representation of the wide variety of sounds associated with seizure and no-seizure activity with the least amount of samples possible.

In an initial exploratory phase of the study, 5 internal non-clinical participants evaluated the developed AI-driven sonification algorithm in several variations to tune various algorithmic hyperparameters. The following variations were considered in this study, with the settings chosen for the external survey outlined in bold:

- Sonification **with** and without an ECG removal algorithm to reduce the interference from ECG;
- Changing the shape of the probability density function from uniform to U-shape versus **no reshaping**;
- **Minimum speed factor** (for seizure-epochs) ranging between **60** and 20;
- Effect of reducing/increasing the **number of EEG channels** from **8** to 2/18, respectively.

After this initial analysis, the best performing variation of the algorithm was evaluated among the clinical interpreters (healthcare professionals, the targeted end-users of this method) using the developed online platform. The results obtained from this initial exploratory phase are included as part of the Supplementary Results.

### Evaluation metrics

The kappa statistic measures the interrater reliability and agreement level between different annotators^59^. While Cohen’s kappa originally measured the interrater reliability between two raters, the Fleiss kappa is an adaptation of Cohen’s kappa for three or more raters^60^. In both cases, the kappa statistic is defined as:

1 $$\kappa = \frac{{p - p_{e} }}{{1 - p_{e} }}$$

where $p$ is the relative observed agreement among raters, measured as the number of instances where raters agree divided by the total number of instances, and $p_{e}$ is the probability of agreement by chance.

The confidence interval of 95% of this measure is given by:

2 $$CI\left( \alpha \right) = \kappa \pm Z_{s} \left( {n,\alpha } \right)\cdot\frac{{SD_{\kappa } }}{\sqrt n }$$

where $Z_{s} \left( {n,\alpha } \right)$ indicates the z-score for $n = 79$ samples (patients) and an estimation error $\alpha = 0.05$. $SD_{\kappa }$ indicates the standard deviation for kappa, given by:

3 $$SD_{\kappa } = \sqrt {\frac{{p\left( {1 - p} \right)}}{{\left( {1 - p_{e} } \right)^{2} }}}$$

The kappa score can range from − 1 to + 1. The level of agreement can be interpreted depending on the kappa value range, from slight (0.01–0.20), fair (0.21–0.40), moderate (0.41–0.60), substantial (0.61–0.80) and almost perfect agreement (0.81–1.00). The kappa score has been previously used in the literature to measure the inter-observer agreement among seizure-raters^20,61^.

While comparing the level of agreement between sets of different raters or annotators, it is important to provide statistical significance in order to show that a certain distribution (in this particular case, given by kappa mean and variance) is sufficiently similar. For this purpose, the Student t-test is used to obtain the t-statistic of the two sample distributions to compare. For the particular case where both distributions have the same number of samples ($n = 79$), the t-statistic reduces to the following expression:

4 $$t = \frac{{\kappa_{1} - \kappa_{2} }}{{\sqrt {\frac{{SD_{{\kappa_{1} }}^{2} + SD_{{\kappa_{2} }}^{2} }}{n}} }}$$

where $\kappa_{i}$ and $SD_{{\kappa_{i} }}$ are the mean and standard deviations of the kappa statistic of each distribution. The *p*-value can then be derived from the *t*-statistic using the inverse of the cumulative density function (CDF) of a *t*-students distribution of n degrees of freedom.

The performance of the sonification algorithm is evaluated at the patient level, meaning that only the presence or absence of seizures is being evaluated. Thus, in order to establish a proper comparison with the AI algorithm alone, a single probabilistic output per patient was obtained by taking the maxima across time, i.e. taking the maximum across EEG channels and time over the smoothed probabilistic output (using the original 60 s moving average filter):

5 $$AI = \mathop {\max }\limits_{t} \left\{ {AI\left[ t \right]} \right\} = \mathop {\max }\limits_{t} \left\{ {\mathop {\max }\limits_{ch} \left\{ {AI\left[ {ch,t} \right]} \right\}} \right\}$$

The area under the receiver-operating curve (AUC ROC)^62,63^ is used to measure the performance of a binary classification AI algorithm. In general, this metric is computed from the predictions given by a model with respect to the ground truth (true value). In this work, the AI-probabilistic output obtained from (5) is used as a prediction, while the majority vote among the three annotators was used as a ground truth. In the best-case scenario, the ROC curve would converge into a squared shape of area equal to one, and worst-case scenario (predictions obtained randomly), the curve would approximate a right triangle of area equal to 0.5 (equivalent to flipping a coin). Thus, the expected AUC score for the AI algorithm would range from 0.5 to 1.

The AUC can also be used as a measure of overlap between two probability distributions—the larger the overlap, the smaller the AUC. In our analysis, it will be used as a measure to quantify how influential a particular EEG characteristic (amplitude, duration) or a system parameter is on the errors or correct answers. For example, in the case of type-II errors (missing seizures), large AUC values would indicate that a particular feature drives the “missed” decision.

## Results

The survey was conducted through the web page with the link disseminated by e-mail invitations. Twenty-three experienced clinicians took part in the survey and answered all audio questions (without any access to the EEG signal). The answers of the survey participants were first contrasted with the experienced annotators who used a full continuous video EEG setup to annotate the signal.

**Figure 4:**
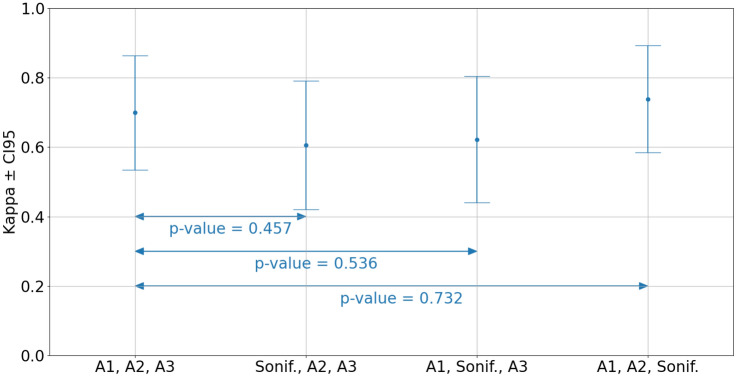
Fleiss kappa while exchanging one annotator by the AI sonification survey results and its p-values.

Figure 4 shows the mean and the 95% confidence interval of the Fleiss’ kappa for inter-rater agreement using any three annotators at each time. The results similarly indicate no statistically significant difference between the accuracy obtained by an experienced EEGer and the healthcare professionals with access to sonified EEG only, with all *p*-values higher than 0.457.

**Figure 5:**
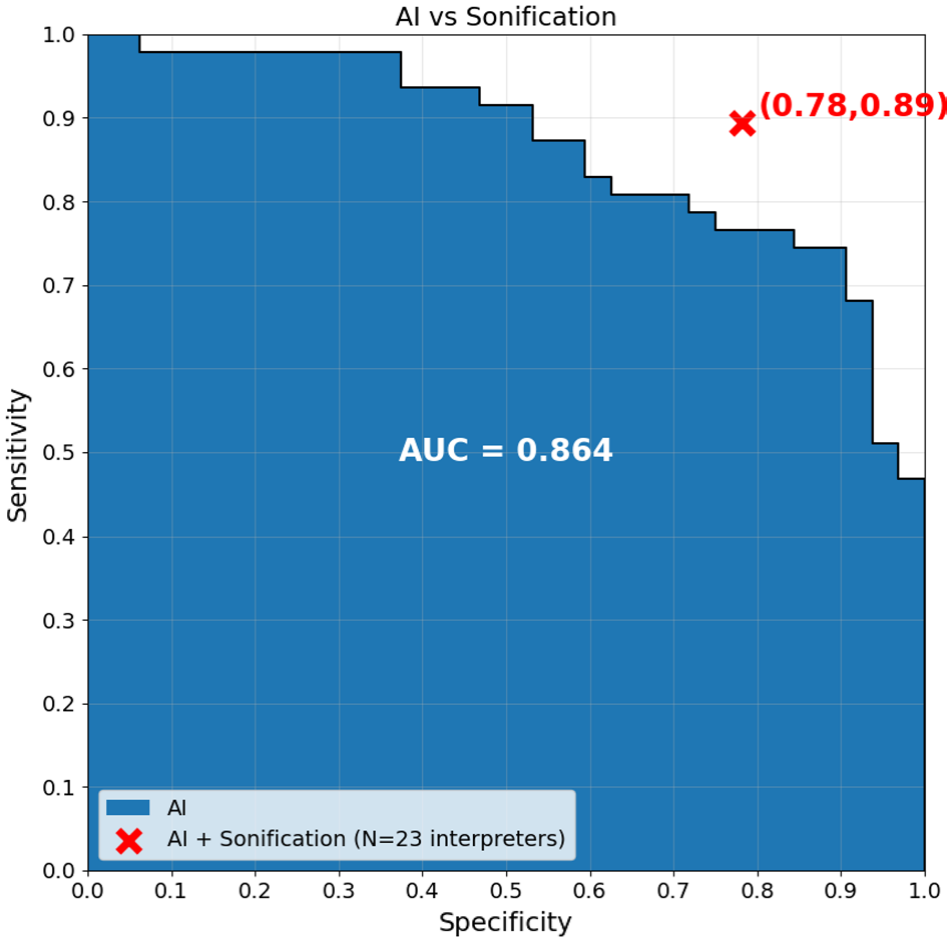
The area under the curve (AUC) for the AI probabilistic output and sensitivity/specificity of the majority vote of the survey participants.

Figure 5 shows the performance obtained from the AI sonification survey compared to the accuracy of the AI algorithm alone in terms of the AUC. The red mark indicates the performance obtained by the 23 clinical interpreters. It can be seen that the performance of AI-driven sonification is superior to any point in the curve of the AI algorithm alone, implying that the additional benefits of sonification and a human interpreter in the loop are advantageous in terms of the accuracy of detecting patients suffering from seizures.

With an intent to have a deeper dive into the analysis of errors obtained with AI sonification, Table 1 shows the confusion matrix of the AI-driven sonification (clinical interpreters only), with a total of seven false alarms (false positives) and five missed seizure patients (false negatives).

**Table 1:** Confusion matrix for the AI sonification majority vote.

Predicted	Actual	
	Positive	Negative
Positive	42	7
Negative	5	25

In order to better understand the source of the errors of the algorithm, an analysis of the most influential factors was conducted based on the results survey to reveal what is the algorithm most sensitive to in terms of characterization of EEG itself and thus the resultant audio. Focusing on false negatives from Table 1, Table 2 shows the statistics of different characteristics of the seizure-present recording in terms of seizure duration, EEG root mean square (RMS) amplitude of the seizures, the number of annotators agreeing, and the AI probabilistic output, to compare detected (42 patients) and undetected patients (5 patients). The AUC scores which are reported for each characteristic can be interpreted as the quantitative measure of importance towards the separation between detected and undetected seizure patients. The missed patients can be mainly attributed to low AI probabilistic output (an AUC of 0.981) and the low amplitude of the EEG (an AUC of 0.861).

**Table 2:** Average ± CI95 for duration, amplitude RMS and average AI probability for correctly detected and missed seizures.

Seizure patients (N = 47)	Duration (s)	Amplitude RMS (μV)	# Annotators agreeing	Seizure probability (AI)
Detected (N = 42)	496 ± 157	67.8 ± 28.2	2.90 ± 0.0925	0.790 ± 0.0746
Missed (N = 5)	279 ± 207	18.6 ± 8.18	2.400 ± 0.629	0.228 ± 0.1058
AUC	0.595	0.861	0.752	0.981

Focusing on false positives from Table 1, Table 3 shows the statistics of different characteristics of the non-seizure patients in terms of the number of annotators disagreeing on the health status and the AI probabilistic output to compare the detected (25 non-seizure patients) and wrongly detected as seizure (5 patients).

**Table 3:** Average ± CI95 for AI probability for correctly detected seizures and missed seizures.

Non-seizure patients (N = 32)	# Annotators disagreeing	Seizure probability (AI)
Detected (N = 25)	0.280 ± 0.188	0.287 ± 0.075
False alarms (N = 7)	0.428 ± 0.477	0.526 ± 0.125
AUC	0.574	0.851

The analysis of the results of all the 23 clinical interpreters revealed that the survey results were stable in 76 out of 79 presented audio samples for the decision, requiring at least two more participants for the decision to be changed between seizure to non-seizure categories. Only 3 of the 79 audio samples were unstable, with 1 or 2 extra votes required to swing the decision to the opposite. In addition, 1/3 of these unstable examples produced a wrong classification after the majority vote, while only 15% of the stable results turned into a wrong classification.

Additional supporting tables and figures are provided as Supplementary Results to compare the following:

- The survey results when conducted over non-clinical interpreters.
- The influence of parameter tunning during the development stage of the system.
- The performance variation as a function of the number of sonified EEG channels.

## Discussion

EEG monitoring is considered the gold standard for detecting seizures in newborns^64^. However, continuous EEG monitoring in clinical settings is limited due to the lack of specialists onsite^15^. In addition, interpretation of EEG is a challenging task, and neurophysiologists are not available 24/7 to the clinical team. In the developing world, expertise availability is even scarcer^65^, especially in the first 24 h after birth.

Without timely neurophysiological support, healthcare professionals must rely on a simplified aEEG version of EEG or clinical manifestations to detect possible brain injuries. Unfortunately, both approaches are inaccurate in detecting seizures^13,18,19,66^. The misdiagnosis does not improve even when continuous electroencephalography is available, but the timely interpretation is not. For example, in the secondary data analysis of 2 European multicenter cohort studies from 2 clinical trials^67^, with continuous EEG monitoring available, 20% of newborns with seizures were misdiagnosed, with some not given anti-seizure medication at all and others given medication before seizures started. Similarly, 258 newborns received anti-seizure medication in a clinical setting, whereas only 154 newborns were confirmed with seizures retrospectively using EEG. From those wrongly diagnosed, 47 infants received multiple anti-seizure medication doses throughout the study.

Timely intervention in reducing the severity of brain damage due to encephalopathy is very important. In the same study^67^, of those who received medication correctly only, 17% received it within 1 h of seizure onset. It was shown that seizures treated within 1 h of seizure onset subsequently had the lowest seizure burden compared with infants who received anti-seizure medication after 1 h of seizure onset.

Scaling up the skills of healthcare professionals in managing asphyxia in newborns has been ranked as the second-highest of research priority for improving newborn health and birth outcomes by 2025^68^.

With the increasing use of prolonged neonatal cEEG monitoring and insufficient neonatal neurophysiology expertise, additional support from automated seizure detection algorithms could be the solution. While several high-accuracy, automated, AI-driven solutions for seizure detection were reported^24,34,51^ to maximize the use and exploit the benefits of such an automated system, the AI models used have to be explainable and keep the medical professional in the loop. These are some of the key requirements for more pervasive use of such tools^69^. Explainable AI is a key enabler that augments the physician’s capabilities without taking away the decision making. By developing human-in-the-loop AI, we may enable an ideal symbiosis of human experts and AI models, exploiting the strengths of both while at the same time overcoming their respective limitations^70,71^.

The discussion of the Supplementary Results can be found in Supplementary Discussion.

### AI sonification versus human annotators

The Fleiss kappa scores presented in Fig. 4 indicate that the accuracy obtained by a human interpreter analysing the sonified 1–2 h segment of EEG is not statistically different from that of the experienced EEG interpreters analysing full multi-channel EEG. The kappa statistics were computed using the asymptotic definition as in^20,59,60^. Bootstrap methods^72,73^ have also been tried in this study to estimate the CI95 of the kappa statistics, with minor differences observed.

In^20^, three experienced EEG experts reviewed over 4000 h of multi-channel EEG in 120 h, which corresponds to the rate of ~ 2 min per hour of EEG. The number of identified neonates with seizures was different for each reviewer, while the agreement on seizure events was only 78%. The AI-driven sonification compressed 1 h of EEG to just 5 s of audio (approximate average across the whole dataset) by focusing on the important segments of EEG (those with seizures), while remarkably achieving the same level of performance with virtually no training required.

One of the main bottlenecks of the more pervasive usage of EEG monitoring systems is the lack of availability of interpretation expertise 24/7. Partly because of that, methods like aEEG aimed to simplify visual interpretation. However, some focal, low-amplitude, and brief seizures may be missed^66^. The general cost of the equipment can be an additional limitation, especially in the developing world. These limitations have triggered the research towards the brain stethoscope project, a low-cost and intuitive solution for quick EEG review^74–76^.

A technological framework to assist the early detection of seizure events is proposed here; acquired EEG data would be analyzed through an always-on AI system that would raise the alarm to notify the clinical staff in the NICU immediately. The presented EEG acoustic interpretation would then be available on-demand for the clinical personnel to review the seizure event (reported by AI) intuitively, with an accuracy proven to be on par with trained EEG experts at detecting seizure patients. That would enable higher confidence for the clinical personnel to call for a full EEG review while reducing the burden of the EEG review process amongst paediatric neurologists.

### AI-driven sonification versus standalone AI

As shown in the previous section, the ground truth for this dataset is subject to interobserver disagreement. However, to further analyse the algorithm performance, a single representation of the ground truth needs to be chosen, and this dataset was represented with consensus annotations across the three neurophysiologists who annotated the EEG.

When considering a human interpreter in an AI-driven decision support system, it is important to quantify the gain obtained by the designed system with respect to the task performed by AI alone. From Fig. 5, it can be seen that there is a clear benefit to having the human in the loop of decision making. The AUC illustrates the performance of the AI in a curve that shows the sensitivity/specificity trade-off for all possible AI probabilistic thresholds. Although the performance is improved with respect to the AI system alone, even a perfectly accurate AI system would require some human verification ultimately, especially in clinical settings. The designed method provides intuitive interpretability to the EEG signal and keeps the human factor in the loop.

DL methods do not always learn intuitively understandable discrimination mechanisms, so they are often referred to as black-box AI systems^77^. Although the general trend for ML and AI is to develop autonomous systems capable of detaching from having a human supervisor in the loop^78^, medicine is exceptional in this regard: understanding how and why a machine decision has been made is essential among medical professionals^79^. In addition to that comes the new European General Data Protection Regulation (GDPR), in which black-box methods will not be allowed to be used in business if they cannot explain why a decision has been made^80^. Here, the paradigm is different; AI is not meant to decide on the data directly but to be used as an attention mechanism to assist doctors’ data interpretation. From this perspective, the proposed system is GDPR-compliant, and it also contributes to making AI more approachable and trusted among medical professionals, improving human–computer interactions.

### Analysis of errors

The accuracy with respect to the consensus ground truth is shown in Table 1 in the form of a confusion matrix, from where a sensitivity (0.89) and specificity (0.78) can be inferred. The reported sensitivity values for detection of seizure presence in a patient for an aEEG, which also aims to simplify standard EEG visual analysis for non-EEG experts in the NICU, ranges from 0.22 to 0.57, with no false positives among the control group^81^. However, the ictal cohort of patients was significantly larger than the control group. Other studies with a more balanced but smaller cohort have shown that aEEG might result in overdiagnosis with respect to standard EEG, with high sensitivities (0.80) but specificities as low as 0.50^82^. Our sound-based solution has been evaluated in a relatively balanced dataset (47 records with seizures vs 32 without any), showing strength in both sensitivity and specificity.

Only five training examples were preselected as training for the users, trying to be sufficiently representative of the diversity of each class (seizure versus background). Still, these may not be sufficient to depict all the possible variations in complex EEG signals. EEG requires a lot of understanding of its underlying characteristics to be accurately interpreted. Previous studies showed that the sensitivity at visually detecting seizure events could be between 2 to 6 times higher in those with previous experience at EEG analysis^83^. In this regard, it is still remarkable that the achieved accuracy with such few training examples could improve further with a wider training set.

#### Misses

The detection of the presence of seizure events can be seen as a function of (a) the duration of the audio that covers seizure events; and (b) the discriminatory perceptual patterns in each seizure event, such as pitch changes and the volume.

The duration of audio, in turn, is a function of the own duration of the seizure events and the AI compression factor, which relies on the ability of AI to detect those events. From Table 2, it can be seen that the duration of the correctly detected seizures is, on average, twice longer than the duration of the missed seizures. The detection of short seizures has been historically challenging for both visual interpretation and automatic detection; the inter-rater agreement is typically lower on short seizures^20^, and the length of the seizure often impacts the performance of the seizure detection algorithms, showing detection rates of 70% for seizures shorter than 1 min versus more than 95% for seizures longer than 4 min^24^.

The role of the amplitude in the misdetection of seizure patients is estimated to be larger than the role of duration in this study, with an AUC of 0.861 compared to 0.595 for the duration. The amplitude of the EEG signals is significantly larger for the detected patients. In this sense, the aEEG has proven to be less effective at detecting low-amplitude seizure events^66^.

The AI probability plays the most significant role in this algorithm as it affects both the duration and the volume, with an AUC of 0.981. It can be seen that it is much lower on the missed patients than that on the detected. While the algorithm relies on AI to start with, it is shown to perform better than AI alone. The perception of the human ear can combine the benefits of being exposed to perceptual seizure characteristics in audio, such as pitch changes and duration, and compensate each other when one fails to convey discrimination.

Finally, more annotators agree on correctly detected seizure patients than missed seizure patients, with an AUC of 0.752. This indicates that some of the errors of the algorithm are subjective, and some annotators might find them to be correct instead of errors.

#### False alarms

It can be seen from Table 3 that in the wrongly detected seizure patients, more annotators disagree than the correctly identified non-seizure patients. However, an AUC of that characteristic is much lower than that of the missed seizure patients from Table 2 (0.574 vs 0.752), indicating that this cannot be considered the main factor in false alarms. The AI probability is lower in the correctly identified non-seizure patients (an AUC of 0.851). Again, the AUC is smaller than that of missed seizures reported in Table 2. False alarms are a product of detecting something abnormal in the audio stream and making a judgment call on whether this abnormality is sufficiently different from the background, often forgetting that the pitch evolution must be present for it to be labelled as a seizure. False alarms can be further decreased with more training examples, which will increase the coverage of heard situations and thus increase the listener’s confidence and competence. In this study, the results are obtained with virtually no training, and only five indicative examples were used for training.

### Limitations of the study

A public dataset of neonatal EEG was utilized in this study. The dataset comprises 79 multi-channel 1–2 h EEG recordings annotated by three experts. The usage of the dataset allows for complete replication of the results obtained in this study, the reproduction of the resultant sounds by other researchers and assessing the level of agreement with other annotators. Certain limitations are inherited with this dataset. The data are preselected. Further study needs to be conducted to assess the performance in a real clinical environment by retrospectively analysing long unedited continuous EEG and mimicking the scenario outlined in this study.

The definition of seizure presence considered in this study was that the EEG contains at least one 10 s-long seizure. However, a more clinically relevant threshold could be used as the definition of seizure presence^84,85^. Thus, the presented results might inflate the number of false positives with respect to the clinical relevant seizures.

The clinical interpreters were comprised of experienced healthcare professionals, primarily neonatologists with 10+ years of experience. Participation in the survey was conducted through personal invitation. Because a particular audience was targeted, the resultant clinical cohort is of moderate size. However, the survey requires a certain time commitment, and healthcare professionals are busier than ever, facing the difficulties brought by the pandemic of COVID-19. During the survey design, many initiatives were researched to limit the listening burden by randomly subsampling the sounds for the survey from the dataset or increasing the speed of sonification to reduce the number of audio questions. These initiatives were found to not significantly shorten the survey duration while posing extra challenges in the analysis of results and test power. In the end, it was agreed to survey all 79 examples. The 23 clinicians who participated in the survey answered all 79 questions. To compensate for the moderate number of participants, the majority vote was utilized to represent the accuracy of the AI-driven sonification. The analysis of the results revealed that 76 out of 79 examples for the decision to be changed between seizure to non-seizure categories indicated that the majority vote label is stable in the vast majority of examples. Also, more unstable examples produced a wrong classification after the majority vote than the stable examples (33% vs 15%), showing that the majority of the errors are concentrated on the non-consensual and possibly less obvious examples.

In the comparison of the AI-driven sonification performance with the EEG annotators, it can be argued that the latter had access to the video and EEG, which allowed for the influence of many observable physiological artifacts whereas those had to be taken care of in an algorithmic manner with the AI-driven sonification.

The study only evaluates the ability of the AI-driven sonification algorithm with the human interpreter to detect the presence or absence of seizures in a given EEG segment of typically 1–2 h duration. The algorithm is not designed to detect individual seizure events or report their duration. The latter is much more critical for prognostication purposes which can be done offline and for which pure AI-based seizure detection algorithms can be used.

### Availability of the sounds, code and AI models

The resultant sounds of the AI-driven sonification algorithm generated from the open-source dataset can be downloaded via github https://github.com/SergiGomezQuintana/EEGsoundSurvey/tree/main/html/audio.

## Conclusions

A new method that allows for acoustic interpretation of complex neonatal EEG brain signals is proposed. A survey has been conducted to assess the level of accuracy of this method among clinical personnel with no EEG interpretation experience. The overall accuracy of detecting the seizure presence in an EEG recording was measured as Sensitivity of 0.89 and Specificity of 0.78, evaluated on twenty-three healthcare professionals (clinical interpreters). This compares favourably with the reported aEEG accuracy for the same task. In fact, the obtained accuracy is comparable with trained EEG interpreters with years of specialized experience. The method facilitates the review of 1 h of EEG in just 5 s, while the time typically spent on EEG visual assessment is 2 min per hour of EEG. The AI-driven solution outperforms the AI algorithm alone by allowing the human interpreter to leverage both the seizure characteristics and AI-driven attention mechanisms. The intuitiveness behind acoustic interpretation allows this method to be used almost off-the-shelf—just five examples were presented to the participants prior to the survey, implying less than 1 min of training for obtaining these results.

The AI-driven sonification shows a great potential to simplify the task of detecting patients suffering from seizures. While the method is mainly oriented toward non-EEG expert medical professionals on the frontline in the NICU, neurologists and neurophysiologists can similarly benefit from acoustic interpretation to speed up visual EEG assessment.

The proposed method serves as a human–computer interface by providing an extra layer of explainability to potentially any AI algorithm. Future research is needed to quantify the added value of acoustical interpretation of EEG as a function of AI algorithm performance.

## Supplementary Information

Supplementary Information 1.

Supplementary Audio 1.

Supplementary Audio 2.

Supplementary Audio 3.

## Data Availability

The datasets analysed during the current study were made publicly available by the Helsinki University Hospital through Zenodo repository under creative commons license, https://doi.org/10.5281/zenodo.2547147.

## References

[1] WHO. Newborns: Improving survival and well-being. World Health Organization. https://www.who.int/news-room/fact-sheets/detail/newborns-reducing-mortality (2020).

[2] Lawn JE, Manandhar A, Haws RA, Darmstadt GL (2007). Reducing one million child deaths from birth asphyxia: A survey of health systems gaps and priorities. Health Res. Policy Syst..

[3] Douglas-Escobar M, Weiss MD (2015). Hypoxic-ischemic encephalopathy: a review for the clinician. JAMA Pediatr..

[4] Delanty N, Vaughan CJ, French JA (1998). Medical causes of seizures. Lancet.

[5] Eriksson M, Zetterström R (1979). Neonatal convulsions incidence and causes in the Stockholm area. Acta Paediatr..

[6] Lanska MJ, Lanska DJ, Baumann RJ, Kryscio RJ (1995). A population-based study of neonatal seizures in Fayette county, Kentucky. Neurology.

[7] Ronen GM, Penney S, Andrews W (1999). The epidemiology of clinical neonatal seizures in Newfoundland: A population-based study. J. Pediatr..

[8] Scher MS, Painter MJ, Bergman I, Barmada MA, Brunberg J (1989). EEG diagnoses of neonatal seizures: Clinical correlations and outcome. Pediatr. Neurol..

[9] McBride MC, Laroia N, Guillet R (2000). Electrographic seizures in neonates correlate with poor neurodevelopmental outcome. Neurology.

[10] Nagarajan L, Palumbo L, Ghosh S (2010). Neurodevelopmental outcomes in neonates with seizures: A numerical score of background encephalography to help prognosticate. J. Child Neurol..

[11] Uria-Avellanal C, Marlow N, Rennie JM (2013). Outcome following neonatal seizures. Semin. Fetal Neonatal. Med..

[12] Oza S, Lawn JE, Hogan DR, Mathers C, Cousens SN (2014). Neonatal cause-of-death estimates for the early and late neonatal periods for 194 countries: 2000–2013. Bull. World Health Organ..

[13] Murray DM (2008). Defining the gap between electrographic seizure burden, clinical expression and staff recognition of neonatal seizures. Arch. Dis. Child Fetal Neonatal. Ed..

[14] Boylan GB, Stevenson NJ, Vanhatalo S (2013). Monitoring neonatal seizures. Semin. Fetal Neonatal. Med..

[15] Boylan GB, Burgoyne L, Moore C, O’Flaherty B, Rennie JM (2010). An international survey of EEG use in the neonatal intensive care unit. Acta Paediatr. Int. J. Paediatr..

[16] Husain AM (2005). Review of neonatal EEG. Neurodiagn. J..

[17] Rakshasbhuvankar A, Paul S, Nagarajan L, Ghosh S, Rao S (2015). Amplitude-integrated EEG for detection of neonatal seizures: a systematic review. Seizure.

[18] Rennie JM (2004). Non-expert use of the cerebral function monitor for neonatal seizure detection. Arch. Dis. Child. Fetal Neonatal. Edn..

[19] Zhang L, Zhou Y-X, Chang L-W, Luo X-P (2011). Diagnostic value of amplitude-integrated electroencephalogram in neonatal seizures. Neurosci. Bull..

[20] Stevenson NJ (2015). Interobserver agreement for neonatal seizure detection using multichannel EEG. Ann. Clin. Transl. Neurol..

[21] Aarabi A, Wallois F, Grebe R (2006). Automated neonatal seizure detection: A multistage classification system through feature selection based on relevance and redundancy analysis. Clin. Neurophysiol..

[22] Temko, A., Thomas, E., Boylan, G., Marnane, W. & Lightbody, G. An SVM-based system and its performance for detection of seizures in neonates. in *Proceedings of the 31st Annual International Conference of the IEEE Engineering in Medicine and Biology Society: Engineering the Future of Biomedicine, EMBC 2009* 2643–2646 (2009) 10.1109/IEMBS.2009.5332807 19963774

[23] Thomas EM, Temko A, Lightbody G, Marnane WP, Boylan GB (2010). Gaussian mixture models for classification of neonatal seizures using EEG. Physiol. Meas..

[24] Temko A, Thomas E, Marnane W, Lightbody G, Boylan G (2011). EEG-based neonatal seizure detection with Support Vector Machines. Clin. Neurophysiol..

[25] Boashash B, Boubchir L, Azemi G (2012). A methodology for time-frequency image processing applied to the classification of nonstationary multichannel signals using instantaneous frequency descriptors with application to newborn EEG signals. Eurasip. J. Adv. Signal Process..

[26] Stevenson NJ (2012). An automated system for grading EEG abnormality in term neonates with hypoxic-ischaemic encephalopathy. Ann. Biomed. Eng..

[27] Mirowski P, Madhavan D, LeCun Y, Kuzniecky R (2009). Classification of patterns of EEG synchronization for seizure prediction. Clin. Neurophysiol..

[28] Truong ND (2018). Convolutional neural networks for seizure prediction using intracranial and scalp electroencephalogram. Neural Netw..

[29] Hussein R, Palangi H, Ward RK, Wang ZJ (2019). Optimized deep neural network architecture for robust detection of epileptic seizures using EEG signals. Clin. Neurophysiol..

[30] Roy Y (2019). Deep learning-based electroencephalography analysis: A systematic review. J. Neural Eng..

[31] O’Shea, A., Lightbody, G., Boylan, G. & Temko, A. Neonatal seizure detection using convolutional neural networks. in *IEEE International Workshop on Machine Learning for Signal Processing, MLSP* vols 2017-Septe 1–6 (2017).

[32] Linardatos P, Papastefanopoulos V, Kotsiantis S (2020). Explainable AI: A review of machine learning interpretability methods. Entropy.

[33] Su J, Vargas DV, Sakurai K (2019). One pixel attack for fooling deep neural networks. IEEE Trans. Evol. Comput..

[34] Ahmed R, Temko A, Marnane W, Lightbody G, Boylan G (2016). Grading hypoxic–ischemic encephalopathy severity in neonatal EEG using GMM supervectors and the support vector machine. Clin. Neurophysiol..

[35] O’Shea, A., Lightbody, G., Boylan, G. & Temko, A. Investigating the Impact of CNN Depth on Neonatal Seizure Detection Performance. In *Annual International Conference of the IEEE Engineering in Medicine and Biology Society.* 2018, pp. 5862–5865 (2018). 10.1109/EMBC.2018.8513617 30441669

[36] Gomez, S. *et al.* On sound-based interpretation of neonatal EEG. In *29th Irish Signals and Systems Conference, ISSC 2018* (2018).

[37] Barrass S, Kramer G (1999). Using sonification. Multimedia Syst..

[38] Laennec, R. & Forbes, J. *A Treatise on the Diseases of the Chest, and on Mediate Auscultation* (1838).

[39] Liberman, I. Y. & Shankweiler, D. Speech, the Alphabet, and Teaching to Read. in *NIS Conference-on the Theory and Practice of Beginning Reading Instruction, Learning Research and Development Center* (1976).

[40] Rose AL, Lombroso CT (1970). Neonatal Seizure states. Pediatrics.

[41] Clancy RR, Legido A (1987). The exact ictal and interictal duration of electroencephalographic neonatal seizures. Epilepsia.

[42] Kitayama M (2003). Wavelet analysis for neonatal electroencephalographic seizures. Pediatr. Neurol..

[43] Purves, D. *et al.* Neuroscience. in Sinauer Associates, 2001.

[44] Olivan J, Kemp B, Roessen M (2004). Easy listening to sleep recordings: Tools and examples. Sleep Med..

[45] Khamis H, Mohamed A, Simpson S, McEwan A (2012). Detection of temporal lobe seizures and identification of lateralisation from audified EEG. Clin. Neurophysiol..

[46] Hermann, T. *et al.* Sonifications for EEG data analysis. in *Proceedings of the 2002 International Conference on Auditory Display* (2002).

[47] Baier G, Hermann T, Stephani U (2007). Event-based sonification of EEG rhythms in real time. Clin. Neurophysiol..

[48] Loui P, Koplin-Green M, Frick M, Massone M (2014). Rapidly learned identification of epileptic seizures from sonified EEG. Front. Hum. Neurosci..

[49] Parvizi J, Gururangan K, Razavi B, Chafe C (2018). Detecting silent seizures by their sound. Epilepsia.

[50] Temko, A., Marnane, W., Boylan, G., O’Toole, J. M. & Lightbody, G. Neonatal EEG audification for seizure detection. in *2014 36th Annual International Conference of the IEEE Engineering in Medicine and Biology Society, EMBC 2014* 4451–4454 (2014). 10.1109/EMBC.2014.6944612 25570980

[51] O’Shea A, Lightbody G, Boylan G, Temko A (2020). Neonatal seizure detection from raw multi-channel EEG using a fully convolutional architecture. Neural Netw..

[52] White DM, Van Cott AC (2010). EEG artifacts in the intensive care unit setting. Neurodiagn. J..

[53] Boll SF (1979). Suppression of acoustic noise in speech using spectral subtraction. IEEE Trans. Acoust. Speech Signal Process..

[54] Flanagan JL, Golden RM (1966). Phase vocoder. Bell Syst. Tech. J..

[55] Laroche J, Dolson M (1999). Improved phase vocoder time-scale modification of audio. IEEE Trans. Speech Audio Process..

[56] Temko, A. Estimation of heart rate from photoplethysmography during physical exercise using Wiener filtering and the phase vocoder. in *Proceedings of the Annual International Conference of the IEEE Engineering in Medicine and Biology Society, EMBS* vols 2015-Novem 1500–1503 (2015). 10.1109/EMBC.2015.7318655 26736555

[57] McGee, R. & Rogers, D. Musification of seismic data. In *International Conference on Auditory Display* 201–204 (2017).

[58] Stevenson NJ, Tapani K, Lauronen L, Vanhatalo S (2019). A dataset of neonatal eeg recordings with seizure annotations. Sci. Data.

[59] McHugh ML (2012). Interrater reliability: the kappa statistic. Biochem. Med..

[60] Fleiss JL (1971). Measuring nominal scale agreement among many raters. Psychol. Bull..

[61] Stevenson N, Lauronen L, Vanhatalo S (2018). The effect of reducing EEG electrode number on the visual interpretation of the human expert for neonatal seizure detection. Clin. Neurophysiol..

[62] Bradley AP (1997). The use of the area under the ROC curve in the evaluation of machine learning algorithms. Pattern Recognit..

[63] Fawcett T (2006). An introduction to ROC analysis. Pattern Recognit. Lett..

[64] Pisani F, Pavlidis E (2018). The role of electroencephalogram in neonatal seizure detection. Expert Rev. Neurother..

[65] Haider BA, Bhutta ZA (2006). Birth asphyxia in developing countries: current status and public health implications. Curr. Probl. Pediatr. Adolesc. Health Care.

[66] Hellström-Westas L, Rosén I, de Vries LS, Greisen G (2006). Amplitude-integrated EEG classification and interpretation in preterm and term infants. NeoReviews.

[67] Pavel, A. M. *et al.* Neonatal seizure management: Is the timing of treatment critical? *J. Pediatr.* (2021) 10.1016/j.jpeds.2021.09.058 PMC9067353 34626667

[68] Yoshida S (2016). Setting research priorities to improve global newborn health and prevent stillbirths by 2025. J. Global Health.

[69] Kundu S (2021). AI in medicine must be explainable. Nat. Med..

[70] Patel BN (2019). Human–machine partnership with artificial intelligence for chest radiograph diagnosis. npj Digit. Med..

[71] Schuller, B., Virtanen, T., Riveiro, M., Rizos, G. & Jing, H. Towards sonification in multimodal and user-friendly explainable artificial intelligence. in *Proceedings of the 2021 International Conference on Multimodal Interaction* (2021).

[72] Hardin P, Shumway J (1997). Statistical significance and normalized confusion matrices. Photogramm. Eng. Remote. Sens..

[73] Zapf A, Castell S, Morawietz L, Karch A (2016). Measuring inter-rater reliability for nominal data—Which coefficients and confidence intervals are appropriate?. BMC Med. Res. Methodol..

[74] Poveda, J., O’Sullivan, M., Popovici, E. & Temko, A. Portable neonatal EEG monitoring and sonification on an Android device. In *Proceedings of the Annual International Conference of the IEEE Engineering in Medicine and Biology Society, EMBS* 2018–2021 (2017) 10.1109/EMBC.2017.8037248 29060292

[75] O’Sullivan, M. *et al.* Neonatal EEG interpretation and decision support framework for mobile platforms. In *Proceedings of the Annual International Conference of the IEEE Engineering in Medicine and Biology Society, EMBS* vols 2018-July 4881–4884 (2018). 10.1109/EMBC.2018.8513231 30441437

[76] Gomez, S. *et al.* An EEG analysis framework through AI and sonification on low power IoT edge devices. In *Proceedings of the Annual International Conference of the IEEE Engineering in Medicine and Biology Society, EMBS* 1–4 (2021). 10.1109/EMBC46164.2021.9630253 34891290

[77] Alain, G. & Bengio, Y. Understanding intermediate layers using linear classifier probes. arXiv preprint arXiv:1610.01644 (2016).

[78] Shahriari B, Swersky K, Wang Z, Adams RP, De Freitas N (2016). Taking the human out of the loop: A review of Bayesian optimization. Proc. IEEE.

[79] Holzinger, A., Biemann, C., Pattichis, C. S. & Kell, D. B. What do we need to build explainable AI systems for the medical domain? arXiv preprint arXiv:1712.09923 (2017).

[80] Sartor G, Lagioia F (2020). The impact of the General Data Protection Regulation (GDPR) on artificial intelligence. Eur. Parliament. Res. Serv..

[81] Shellhaas RA, Soaita AI, Clancy RR (2007). Sensitivity of amplitude-integrated electroencephalography for neonatal seizure detection. Pediatrics.

[82] Evans E, Koh S, Lerner JT, Sankar R, Garg M (2010). Accuracy of amplitude integrated EEG in a neonatal cohort. Arch. Dis. Childh. Fetal Neonatal. Edn..

[83] Abend NS, Dlugos D, Herman S (2008). Neonatal seizure detection using multichannel display of envelope trend. Epilepsia.

[84] Clancy R (2006). Summary proceedings from the neurology group on neonatal seizures. Pediatrics.

[85] Pressler R, Boylan G, Marlow N, Blennow M (2015). Bumetanide for the treatment of seizures in newborn babies with hypoxic ischaemic encephalopathy (NEMO): an open-label, dose finding, and feasibility. Lancet Neurol..

