# Supplementary material for: A method for AI assisted human interpretation of neonatal EEG
Source: Sci Rep. 2022 Jun 29;12:10932. doi: 10.1038/s41598-022-14894-4 (PMC9243143; doi:10.1038/s41598-022-14894-4)
Supplement: Supplementary file 1 — Supplementary Information 1. [file 41598_2022_14894_MOESM1_ESM.docx]

A method for AI-assisted human interpretation of neonatal EEG

Sergi Gomez-Quintana^1*^, Alison O’Shea^2^, Andreea Factor^3^, Emanuel Popovici^1^, Andriy Temko^1^

^1^ Electrical and Electronic Engineering, University College Cork, Ireland

^2^ Department of Computer Science, Munster Technological University, Ireland

^3^ Department of Anatomy and Neuroscience, University College Cork, Ireland

[sgomez@umail.ucc.ie](mailto:sgomez@umail.ucc.ie)

Abstract—

The study proposes a novel method to empower healthcare professionals to interact and leverage AI decision support in an intuitive manner using auditory senses. The method's suitability is assessed through acoustic detection of the presence of neonatal seizures in electroencephalography (EEG). Neurophysiologists use EEG recordings to identify seizures visually. However, neurophysiological expertise is expensive and not available 24/7, even in tertiary hospitals. Other neonatal and pediatric medical professionals (nurses, doctors, etc.) can make erroneous interpretations of highly complex EEG signals. While artificial intelligence (AI) has been widely used to provide objective decision support for EEG analysis, AI decisions are not always explainable. This work developed a solution to combine AI algorithms with a human-centric intuitive EEG interpretation method. Specifically, EEG is converted to sound using an AI-driven attention mechanism. The perceptual characteristics of seizure events can be heard using this method, and an hour of EEG can be analysed in five seconds. A survey that has been conducted among targeted end-users on a publicly available dataset has demonstrated that not only does it drastically reduce the burden of reviewing the EEG data, but also the obtained accuracy is on par with experienced neurophysiologists trained to interpret neonatal EEG. It is also shown that the proposed communion of a medical professional and AI outperforms AI alone by empowering the human with little or no experience to leverage AI attention mechanisms to enhance the perceptual characteristics of seizure events.

Keywords— Computational methods, signal processing, medical data, analysis, visualisation, sonification, electroencephalogram, neonates, seizures, deep learning, explainable artificial intelligence

# Supplementary Methods: AI-driven Sonification Algorithm

This section is dedicated to providing an in-detail description of each subroutine of the proposed AI-assisted sonification algorithm, shown in Figure 1 of the main manuscript.

## DSP block: Preprocessing

The signal pre-processing stage applies a set of deterministic operations to adequate the EEG signals before it enters the sonification algorithm’s subsequent stages, reducing the noise level outside the band of interest and EEG artifacts [1]. First, a combination of highpass and lowpass filters removes undesired components outside the frequency range between 0.5Hz and 16Hz. A notch filter removes the interfering 50Hz-AC component caused by the power supply of the acquisition system. Although this component is already considerably attenuated by the lowpass filter, in some cases, the 50Hz component may remain non-negligible and could potentially affect terms of aliasing when downsampling the signal in further stages of the algorithm.

The AC component is not the only potential source of interference when dealing with EEG signals. The electrical activity of the heart often impacts EEG recordings too. It is captured similarly to EEG (by placing electrodes on the skin), and the recorded electrical signal is known as an electrocardiogram (ECG). While EEG signals are usually in the range of tens to hundreds of microvolts (µV), the ECG signals have wider amplitudes in the range of millivolts (mV), meaning that despite good isolation between ECG and ECG EEG electrodes, interference from ECG into EEG can still occur. Furthermore, the ECG frequencies may fall in the same range as neonatal EEG and thus produce seizure-like patterns in EEG signals.

When the ECG signal is provided additionally to the EEG data, it can be used to estimate the amount of ECG interference in any given EEG signal. The following parametric model has been considered in this study: the ECG signal is superposed in the EEG signal as an attenuated and delayed version of the ECG reference.

|  | $s\left[ t \right]=\hat{s}\left[ t \right]+\alpha\cdot ECG\left[ t-\delta\right]$ | (1) |
| --- | --- | --- |

where $s\left[ t \right]$ represents the EEG signal corrupted by ECG, $\hat{s}\left[ t \right]$ the estimation of true EEG signal, and *α,* *δ* are the model parameters for the attenuation delay, respectively. Those are estimated through the location of the maximum correlation between EEG and ECG reference. Once the parameters ($\alpha, \delta$) are obtained, the estimation of the cleaned EEG, $\hat{s}\left[ t \right]$, can be made by Eq. 1. The ECG interference amount ($\alpha$) have temporal variations over long EEG signals. Therefore, the parameters of this model are re-estimated every 8s for 16s segments of EEG. The example of ECG-corrupted and cleaned EEG is shown in Figure S1.

Finally, EEG signals may have big bursts due to electrode disconnections or other types of artefacts, but seizures typically fluctuate in the range of ±100µV. Thus, all signals are normalized to this value for the subsequent sonification states. After the signal normalization, a dynamic-range compressor is applied to reduce all values above ±1 to ensure the signal is contained in this normalized amplitude range.


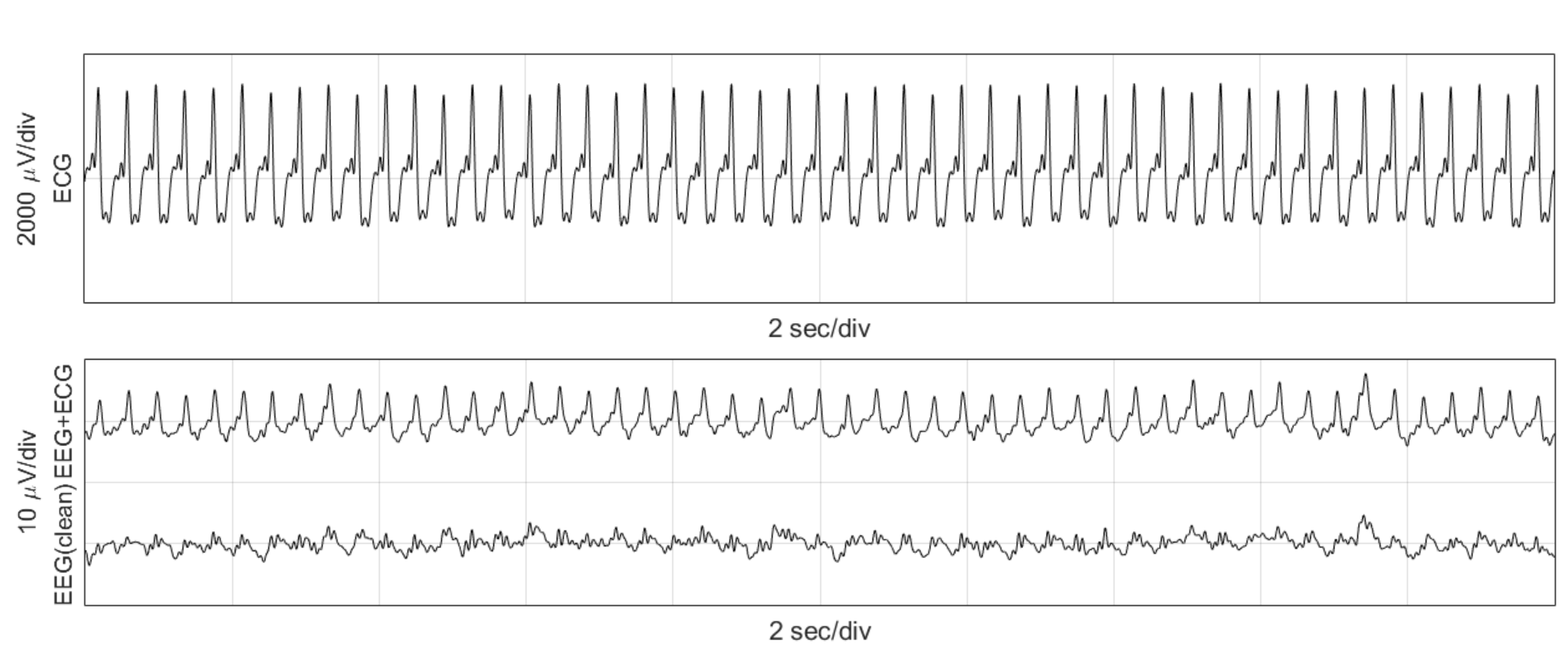


Figure S1. From top to bottom: Recorded ECG signal, recorded EEG (single channel) and cleaned EEG with ECG artifact removed.

## DSP block: Denoiser

A variant of spectral subtraction utilized in speech processing [2] is used to reduce the amplitude of those EEG sections that do not contain seizure events. The short-time Fourier transform (STFT) is computed for the whole EEG signal $X\left[ n,k \right]=STFT\left\{ x\left[ t \right] \right\}$. In equation (2), a spectral profile of the background sections $T[k]$ is estimated as a weighted power spectral density $P_{XX}\left[ n,k \right]=\left| X\left[ n,k \right] \right|^{2}$ with respect to the complementary value of the seizure probability given by the AI algorithm $AI[n]\in\left[ 0,1 \right]$ (i.e. background EEG contributes more to the spectral profile estimation than the seizure events).

|  | $T\left[ k \right]=\sum_{n} P_{XX}\left[ n,k \right]\frac{1-AI[n]}{\sum_{n} \left( 1-AI[n] \right)}$ | (2) |
| --- | --- | --- |

Then, the following gain function is applied to the STFT:

|  | $G\left[ n,k \right]=\left\{ \begin{matrix} 1 & if P_{XX}\left[ n,k \right]\geq T\left[ k \right] \\ \frac{P_{XX}\left[ n,k \right]}{T\left[ k \right]} & otherwise \end{matrix} \right.$ | (3) |
| --- | --- | --- |

Note that the gain function $G\left[ n,k \right]$ has continuity when $P_{XX}=T$, and also $G\left[ n,k \right]\leq1$, meaning that $G\left[ n,k \right]$ actually serves as an attenuation factor. Intuitively, each STFT bin would be attenuated proportionally to its own power when this is smaller than the average power of the background spectral profile ($T$). The denoised EEG STFT ($Y$) is obtained as:

|  | $Y\left[ n,k \right]={10}^{AI[n]-1}\cdot G\left[ n,k \right]\cdot X\left[ n,k \right]$ | (4) |
| --- | --- | --- |

The first term adds an additional attenuation factor of up to 10% when seizure probability approaches zero (i.e. no seizure). Finally, the Inverse-STFT is computed to obtain the time-domain denoised EEG signal.

By using this process, any activity that deviates from the average activity in the recording is emphasized by taking into account the level of seizure-ness of such activity.

## DSP block: Phase vocoder

#### Phase vocoder basics

The phase vocoder (PV) is a well-known technique to manipulate signals in the time domain by preserving their spectral properties. The PV was firstly developed to stretch speech signals in time for encoding voice data (hence the term vocoder) [3]. Although this technique was first applied to speech, it also has been used to manipulate the various type of audio [4] and non-audio signals [5, 6]. The PV interpolates new frames between the existing frames of the STFT of the input signal (Figure S2).

The interpolation of the STFT frames is performed on both the magnitude and phase, as $STFT\left\{ \cdot\right\}\mathbb{:C\to}\mathbb{C}^{2}$. While the magnitude can be trivially interpolated by any method (linear, quadratic, spline, etc.), the main challenge on the PV is the phase interpolation (as its name implies). The original implementation [3] ensured that the phase difference (instantaneous frequency) between each frame of the output STFT was the same as the difference between the analogous frames in the input STFT.

Let $X\left[ n,k \right]=STFT\left\{ x\left[ t \right] \right\}$ be the 2-dimensional input *STFT*, where $0\leq t\leq T-1$ indicates the time index of the time-series signal, and $0\leq n\leq N-1$ and $0\leq k\leq K-1$ stand for the 2D indexes of the STFT matrix, being $N$ the total temporal frames of the STFT (columns) and *K* the total number of frequency-bins (rows).

Let $Y\left[ m,k \right]$ be the STFT resulting from the PV algorithm, with $0\leq m\leq M-1$, being $M$ the number of frames of the resulting STFT, which is related to the original number of frames $N$ through the chosen stretching ratio
$R=M/N$. The frame index relation of $m$ with respect to $n$ can be expressed as $m=\left\lfloor R\cdot n \right\rfloor$, and vice versa $n=\left\lfloor\frac{m}{R} \right\rfloor$, where $\left\lfloor\cdot\right\rfloor$ denotes the closest smallest integer. The stretching ratio is defined as positive ($0<R<\infty$), but when $R<1$ the signal would be contracted instead of stretched.

The magnitude of the resulting STFT $\left\| Y\left[ m,k \right] \right\|$ can be obtained through linear interpolation of the input $\left\| X\left[ n,k \right] \right\|$. This can be written using a recurrent expression, where the magnitude of the next frame $\left\| Y\left[ m+1,k \right] \right\|$ is obtained from the previous $\left\| Y\left[ m,k \right] \right\|$ and $\left\| X\left[ n,k \right] \right\|$:

|  | $\left\Vert Y\left[ m+1,k \right] \right\Vert=\left\Vert Y\left[ m,k \right] \right\Vert+\frac{1}{R}\frac{d}{dn}\left\Vert X\left[ n,k \right] \right\Vert$ | (5) |
| --- | --- | --- |

where $\left\| Y\left[ 0,k \right] \right\|=\left\| X\left[ 0,k \right] \right\|$ and $\frac{d}{dn}\left\| X\left[ n,k \right] \right\|=\left\| X\left[ n+1,k \right] \right\|-\left\| X\left[ n,k \right] \right\|$ it is defined as the magnitude difference between two consecutive frames (i.e. discrete derivative).

The phase can be obtained using a similar recurrent equation:

|  | $∡Y\left[ m+1,k \right]=∡Y\left[ m,k \right]+\frac{d}{dn}∡X\left[ n,k \right]$ | (6) |
| --- | --- | --- |

where $∡$ denotes the phase (angle), $∡Y\left[ 0,k \right]=∡X\left[ 0,k \right]$, and $\frac{d}{dn}∡X\left[ n,k \right]=∡X\left[ n+1,k \right]-∡X\left[ n,k \right]=\omega_{X}\left[ n,k \right]$ is the phase difference between two adjacent input frames, and also the instantaneous frequency. Note that in this case, the last term is not scaled by the stretching ratio $R$. This is to keep the phase consistency between adjacent frames.

Rearranging the terms from (6), and considering that $\frac{d}{dn}∡X\left[ n,k \right]=\omega_{X}\left[ n,k \right]$ and $\frac{d}{dm}∡Y\left[ m,k \right]=\omega_{Y}\left[ m,k \right]$ (i.e. the time-derivative of the phase is the instantaneous frequency), one arrives at the following conclusion:

|  | $\omega_{Y}\left[ m,k \right]=\omega_{X}\left[ n,k \right]$ | (7) |
| --- | --- | --- |

In other words, the instantaneous frequency is preserved, which reflects better the PV intentions of stretching the signal in time only without affecting its frequency content.

Finally, the time-domain stretched signal is obtained by the inverse STFT $y\left[ t \right]={STFT}^{-1}\left\{ Y\left[ m,k \right] \right\}$

After this process, if the same sampling frequency is kept on the obtained signal, $y[t]$, as in the original, $x[t]$, it would also keep the same spectral properties (i.e. same frequency content) proportionally stretched in time by a certain factor $R$ as intended. However, the PV can also be exploited to shift the spectra of a signal while keeping its duration when choosing a sampling frequency $R$ times larger than the original. In this case, the stretching ratio can also be expressed as a function of the relationship between input/output sampling frequencies as follows:

|  | $R={{Fs}_{out}}/{{Fs}_{in}}$ | (8) |
| --- | --- | --- |

In this work, the following properties of the phase vocoder are exploited – first, to conventionally raise the spectral characteristics of EEG signals into an audible range; second, to compress the signals in time to make them shorter and, therefore, quicker to listen.

#### Wrapped versus unwrapped phases

As described in [7], the phase of a complex number $∡$ has a cyclic characteristic around $2\pi$, and it is usually reduced to the range $ϴ=\left( \left. -\pi,\pi\right] \right.$ radians, known as the wrapped phase. One can achieve this by adding or subtracting multiples of $2\pi$ until the value fits inside $ϴ$ range. Mathematically speaking, the unwrapped phase can be any real number $∡\in\mathbb{R}$, and the wrapped phase is restricted to $\left( \left. -\pi,\pi\right] \right.$ interval $∡\inϴ$. The wrapped phase can be computed from the unwrapped phase as:

|  | $\left( ∡a \right)_{ϴ}=mod\left\{ \left( ∡a \right)_{\mathbb{R}}+\pi,2\pi\right\}-\pi=\left( ∡a \right)_{\mathbb{R}}-2\pi\left\lfloor\frac{\left( ∡a \right)_{\mathbb{R}}+\pi}{2\pi} \right\rfloor$ | (9) |
| --- | --- | --- |

When dealing with a single complex value series, the phase may convey the total number of revolutions (unwrapped) or just the fractional part over $2\pi$ (wrapped). However, when working with complex sequences, the wrapped phase can lead to inconsistent phase differences and consequently inconsistent instantaneous frequency estimations for the case of the PV. Thus, the unwrapped version of the phase should be used to estimate the instantaneous frequency. The unwrapped phase is obtained by differentiating the wrapped phase, wrapping the difference and integrating it back:


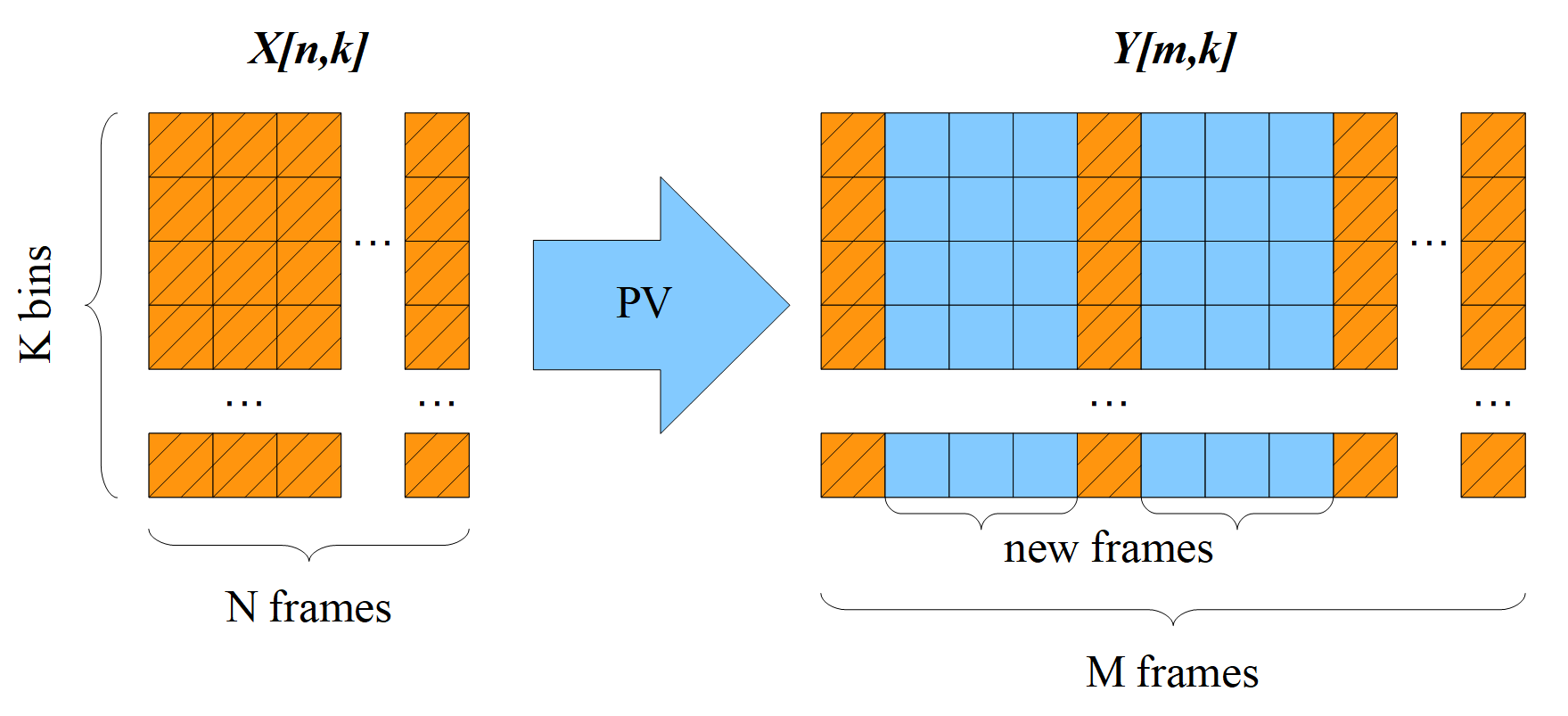


Figure S2. General scheme for input STFT X[n,k] and phase vocoder output STFT Y[m,k].

|  | $\left( ∡S_{n} \right)_{\mathbb{R}}=\sum_{k=0}^{n} \left[ \frac{d}{dk}\left( ∡S_{k} \right)_{ϴ} \right]_{ϴ}$ | (10) |
| --- | --- | --- |

#### Linear frequency estimation

The linear interpolation of the phase leads to a propagated version of the instantaneous frequency $\omega_{X}\left[ n,k \right]$for $\left\lfloor R\cdot n \right\rfloor\leq m<\left\lfloor R\cdot(n+1) \right\rfloor$, which causes a potential discontinuity at $m=\left\lfloor R\cdot(n+1) \right\rfloor$ as the instantaneous frequency changes suddenly from $\omega_{X}\left[ n,k \right]$ to $\omega_{X}\left[ n+1,k \right]$, and ${\omega_{X}\left[ n,k \right]\neq\omega}_{X}\left[ n+1,k \right]$ in general. This issue is not relevant for most of the applications of the PV where the stretching factor may be larger than one but not much, like $R<2$ or $R<4$ at most. However, when $R\gg1$ (i.e. $R=100$), the effect of the discontinuity of the instantaneous frequency becomes more evident to the ears, which will be perceived as sudden tonal jumps.

A better estimation of the instantaneous frequency at each output frame is proposed here through the means of linear interpolation of $\omega_{X}\left[ n,k \right]$ to ensure a continuous (smooth) transition between instantaneous frequencies of consecutive frames $(n,n+1)$:

|  | $\omega_{Y}\left[ m,k \right]=\left( 1-\frac{m}{R}+n \right)\omega_{X}\left[ n,k \right]+\left( \frac{m}{R}-n \right)\omega_{X}\left[ n+1,k \right]$ | (11) |
| --- | --- | --- |

By rearranging terms, it is obtained:

|  | $\omega_{Y}\left[ m,k \right]=\omega_{X}\left[ n,k \right]+\left( \frac{m}{R}-n \right)\frac{d}{dn}\omega_{X}\left[ n,k \right]$ | (12) |
| --- | --- | --- |

which leads to an updated version of the original expression in (6), with an additional term:

|  | $∡Y\left[ m+1,k \right]=∡Y\left[ m,k \right]+\frac{d}{dn}∡X\left[ n,k \right]+\left( \frac{m}{R}-n \right)\frac{d^{2}}{dn}∡X\left[ n,k \right]$ | (13) |
| --- | --- | --- |

As a result of enforcing first-order continuity to the instantaneous frequency with equation (11), the phase $∡Y\left[ m,k \right]=\sum_{i=0}^{m} \omega_{Y}\left[ i,k \right]$ has subsequently second-order continuity.

#### Variable stretching ratio

So far, it has been assumed that the stretching ratio $R$ is constant. But what if we want the *R* to be dynamic depending on the signal properties? For example, suppose that the input STFT is to be stretched by three different stretching factors $R_{1}$, $R_{2}$ and$R_{3}$. One could simply divide the input STFT into those three chosen parts and apply the PV consecutively using $R_{1}$, $R_{2}$ and$R_{3}$, respectively. An example is shown in Figure S3.a, where the input STFT consists of 3 consecutive tones at 8, 16 and 32Hz of 10 seconds long each. A time-dependent stretching factor R affects the output STFT differently; the duration of the first tone becomes twice as larger as in the input (R=2). The second tone remains the same length (R=1). The length of the third tone is halved (R=0.5).

By taking this approach to the limit, one could consider that each input frame $n$ has a well-defined value of $R[n]$ and make use of the PV equations (5) and (13) to extrapolate the magnitude and phase of the output STFT $Y\left[ m,k \right]$ for a given $X\left[ n,k \right]$. Figure S3.b, shows a chirp signal being stretched continuously under this principle.

By doing that, we can achieve a variable-speed PV (VSPV) since the stretching ratio is inversely proportional to the "speed" ($VS[n]$), while also raising the frequency content:

|  | $R\left[ n \right]=\frac{{{Fs}_{out}}/{{Fs}_{in}}}{VS[n]}$ | (14) |
| --- | --- | --- |

Where in particular, the EEG signals in this work need to be raised from a sampling frequency of ${Fs}_{in}=32Hz$ to the audio sampling frequency ${Fs}_{out}=16000Hz$.

Equation (14) allows for decoupling the total stretching ratio into (a) the amount of frequency raising required (numerator) and (b) the actual temporal compression (denominator). The variable speed $VS[n]$ in the VSPV is used in EEG sonification to reduce the reviewing effort time, regardless of the input/output sampling frequencies. AI is used to control the speed factor $VS[n]$, which follows the exponential rule on the given equation:

|  | $VS[n]=V_{max}\left( \frac{V_{min}}{V_{max}} \right)^{AI[n]}$ | (15) |
| --- | --- | --- |

where $V_{max}=3600$, (1 hour compressed of EEG compressed as 1 second of audio for background sections) and $V_{min}=60$ (1 minute of EEG compressed as 1 second of audio for seizure sections) are the maximum and minimum velocities (speeds), $AI[n]\in\left[ 0,1 \right]$.

Given the variable speed, the total duration, $T_{o}$, of the output signal can be precomputed beforehand as a function of the total duration of the input signal, $T_{i}$, and $VS[n]$:

|  | $T_{o}=T_{i}\frac{1}{N}\sum_{n=0}^{N-1} VS[n]$ | (16) |
| --- | --- | --- |

Moreover, the expansion and compression rates ($ER$, $CR$) are defined as the ratio of those durations:

|  | $ER=\frac{1}{CR}=\frac{T_{o}}{T_{i}}=\frac{1}{N}\sum_{n=0}^{N-1} VS[n]$ | (17) |
| --- | --- | --- |

where $T_{i}, T_{o}$ can be measured either in seconds or the number of STFT frames. In a typical use-case of this work, the duration of the obtained signal would often be shorter than the original ($T_{o}<T_{i}$) the $CR$ is frequently utilized to measure the capacity of condensing EEG data into sound.


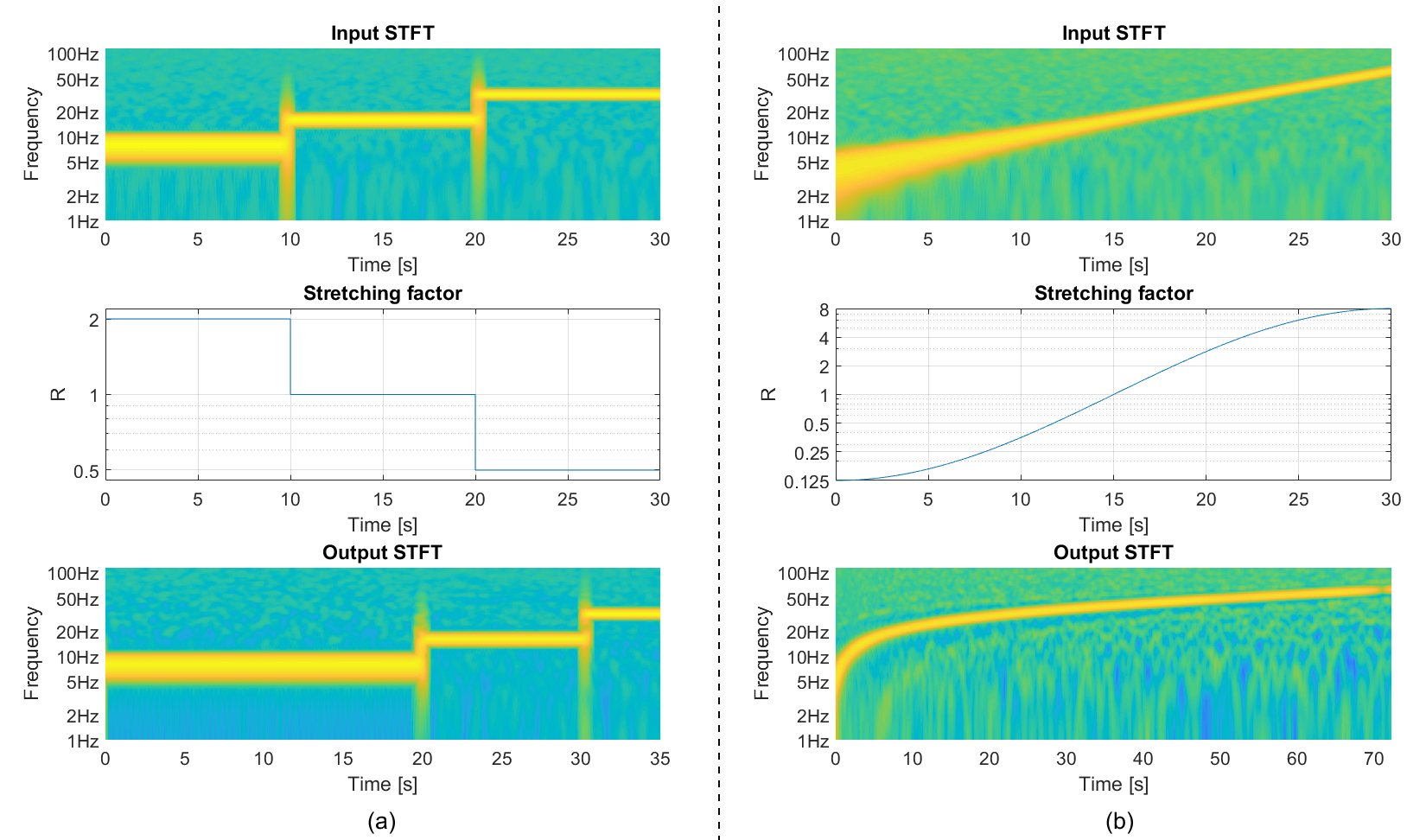


Figure S3. VSPV examples. (a) Tones of 8, 16 and 32Hz stretched/contracted individually. (b) Chirp signal being stretched continuously.

## AI block: Seizure detection model

The seizure probability obtained from an AI algorithm is used in two of the stages of the proposed sonification algorithm: (a) Denoiser and (b) VSPV. Any seizure detection algorithm that provides a probabilistic output per segment and channel of EEG could potentially be used. In this work, the deep learning neonatal seizure detection algorithm is utilized [8]. The model consists of a fully convolutional neural network (FCNN) architecture, which runs on top of raw EEG data and does not require hand-crafted features. The algorithm has been extensively evaluated to show the stage-of the-art generalization error and was also validated on the publicly available Helsinki dataset (same dataset used in this work), showing an area under the curve performance of 0.956 at detecting seizure events.

The FCNN algorithm takes 8s-long fragments of the pre-processed multichannel EEG signal, which is filtered and downsampled to 32Hz, similar to the pre-processing stage in this study. For each segment, the model then generates a probabilistic output per channel. For this study, an overlap of 7s is used to achieve a seizure-probability throughput of 1Hz. By doing so, one probabilistic output per second is given in the form of a multichannel time series. After that, the probabilities are smoothed in each channel and merged into an overall probabilistic output by taking the maximum probability across channels:

|  | $AI\left[ n \right]=\max_{CH} \left\{ AI\left[ ch,n \right] \right\}$ | (18) |
| --- | --- | --- |

Originally [8], a moving-average filter of 60s was used to smooth the probabilistic output in order to balance the rate of false alarms with the rate of detected events to maximize the accuracy of seizure detection. In this work, to allow more events (false and correct) to pass through the smoothing filter, a 15s median filter was utilised to allow more short seizure events in seizure sonification. The smoothed probabilities are used in the denoising stage and in the VSPV where the overall probability obtained from Eq. (18) is combined with the geometric transformation from Eq. (15) to be used to control the speed factor.

## DSP block: Stereo mixer

Up to this point, the signals for each EEG channel have been converted into sound independently, obtaining an audio signal for each EEG channel. Those audio signals need to be mixed into a standard stereophonic sound system so most standard devices can play it. In other words, several independent sources of information (audio from EEG channels) need to be mapped into 2 sources, representing the left and right audio channels. There are many ways to achieve this purpose, depending on the degree of complexity of the model utilized to undertake this goal. Perhaps the most straightforward approach is to map the left/right hemisphere channels into left/right stereo channels, respectively [43]. However, if the listener loses one of the stereo channels, half of the EEG information is lost too. From this perspective, an approach that utilizes the stereo attribute as a complementary but non-essential part of the auditory display is preferred. More complex models adequately approximate the so-called head-related transfer function (HRTF) [52]. These models aim to mimic how humans perceive sound sources as a function of the relative distance, azimuth, elevation and frequency [53, 54]. With HRTF models, the lack of one stereo channel does not suppress the sound source completely. However, the degree of complexity in such cases is unnecessary or even unwanted for the scope of this work. First, due to the attenuation of propagation proportional to the squared of the source distance, some channels would be perceived louder than others, and in this work, all EEG channels are considered equally important. Second, the EEG channels can be displayed in a 2D plane, disregarding the elevation dimension. Lastly, the transfer function to mimic front/back works well for well-known, and natural sounds such as speech, but the listener is a priori not familiarized with sonified EEG. Thus, the main takeaway from these works is the azimuthal dimension. This allows for the localization of the sound source direction [55], thus enabling the listener to differentiate from left, central or right hemisphere sonified electrode pairs.


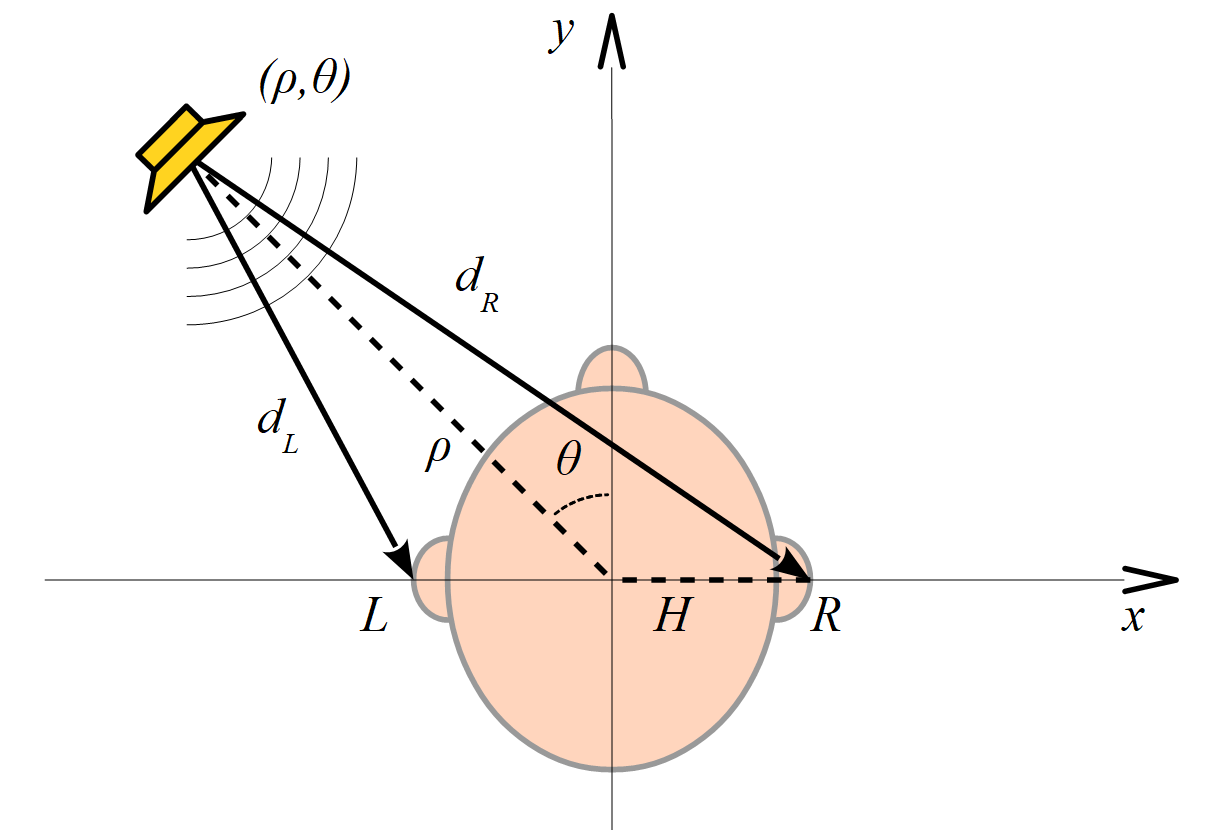


Figure S4. Precedence effect due to interaural delay.

A simple model based exclusively on the precedence effect due to the interaural time delay (ITD) is used to accomplish this goal by virtually situating each audio source in a 2D plane in relation to an imaginary listener [56]. The model assumes a listener with right and left ears (*L* and *R*) separated by a certain opposed distance $H$ from the origin of coordinates (head centre) and that the sound source is at a distance $\rho$ within a certain azimuthal angle $\theta$ defined positive towards left (Figure S4). The model assumes the simplest approach where there are no obstacles between the sound source and the ears (i.e. no attenuation) and that the sound reaches the ears by direct propagation only, with no reflections.

Under this assumption, one can obtain through trigonometry the equations for the left and proper propagation delays $d_{L/R}$ as:

|  | $d_{L/R}=\frac{1}{c}\sqrt{\rho^{2}+H^{2}\pm\rho\cdot H\cdot sin\theta}$ | (19) |
| --- | --- | --- |

where the positive sign is considered for left ($L$). The constant, $c$, indicates the propagation speed of sound in air, which is about 340m/s, and $H$ indicates the radius of the head, which was rounded to 10cm.


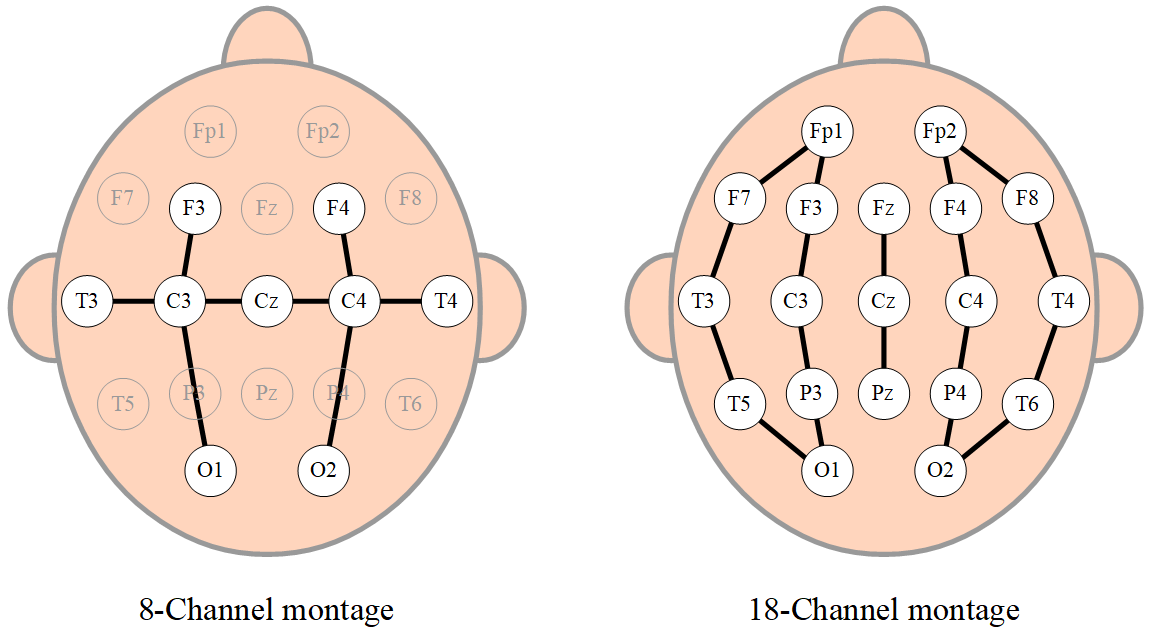


Figure S5. Approximate locations on the scalp of the 8 and 18 channel montages for EEG acquisition.

Under this principle, each audio signal corresponding to each EEG channel is then virtually placed on a specific set of coordinates, $\left( \rho,\theta\right)$, to obtain a pair of delays, $d_{L/R}$, associated with each channel (Table S1). These positions were chosen to be proportional to the actual physical positions (same $\theta$ with augmented$\rho$) of the electrode pairs in the EEG montages utilized in this study: 8-channel and 18-channel montages shown in Figure S5.

Once these delays are obtained, the stereo audio is obtained by applying the corresponding L/R delays to each channel and summing them up onto each stereo channel.

|  | $s_{L/R}\left[ t \right]=\sum_{ch=1}^{N_{CH}} a\left[ t-{d[ch]}_{L/R},ch \right]$ | (20) |
| --- | --- | --- |

where $a\left[ t,ch \right]$ is the time-series matrix of the sonified data, and $s_{L/R}\left[ t \right]$ the left/right stereo audio.

Table S1. Polar coordinates of electrodes (left hemisphere only).

| 8-Channel: | | | | | | | | | | | | |
| --- | --- | --- | --- | --- | --- | --- | --- | --- | --- | --- | --- | --- |
| Chan. | F3–C3 | | | C3–O1 | | | T3–C3 | | | C3–Cz | | |
| $\rho/H$ | 5.2 | | | 6.3 | | | 7.4 | | | 2.6 | | |
| $\theta$ | 60º | | | 140º | | | 90º | | | 90º | | |
| 18-Channel: | | | | | | | | | | | | |
| Chan. | Fp1–F3 | F3–C3 | C3–P3 | | P3–O1 | Fp1–F7 | F7–T3 | T3–T5 | T5–O1 | | Fz–Cz | Cz–Pz |
| $\rho/H$ | 8.0 | 5.1 | 5.1 | | 8.0 | 9.5 | 9.6 | 9.6 | 9.5 | | 2.4 | 2.4 |
| $\theta$ | 30º | 60º | 120º | | 150º | 35º | 70º | 110º | 145º | | 0º | 180º |

# Supplementary Results

Table S2 shows the kappa statistics for inter-rater agreement among four annotators. The first three are original EEG annotators from the dataset, whereas the majority vote represents the fourth annotator among all survey participants. The mean and the 95% confidence intervals are computed for Cohen’s kappa scores. The level of agreement between the three original annotators and the sonification is no different from the agreement level among the three original annotators.

Table S2. Cohen’s kappa scores for annotator pairs and AI sonification algorithm (Kappa ± CI95).

|  | Annotator 1 | Annotator 2 | Annotator 3 | AI sonification |
| --- | --- | --- | --- | --- |
| Annotator 1 | 1 | 0.819±0.130 | 0.651±0.176 | 0.710±0.161 |
| Annotator 2 | 0.819±0.130 | 1 | 0.628±0.180 | 0.686±0.166 |
| Annotator 3 | 0.651±0.176 | 0.628±0.180 | 1 | 0.503±0.205 |
| AI sonification | 0.710±0.161 | 0.686±0.166 | 0.503±0.205 | 1 |

Table S3 presents the performance of AI sonification as a function of various parameters of the system during the development stage when evaluated using the internal survey of 5 participants before the parameters were set and released to the external survey. The baseline setting consists of no ECG removal, no probability function transformation and min speed set to 60 (seizure events are compressed by a speed factor of 60).

Table S3. The influence of the parameters on the performance of AI sonification, in terms of sensitivity, specificity and compression rate.

| Setting | Sensitivity | Specificity | Compression Rate |
| --- | --- | --- | --- |
| Baseline | 0.79 | 0.95 | 727.4 |
| ECG removal | **0.83** | **0.95** | 727.4 |
| Uniform distribution probabilities | 0.81 | 0.86 | 359.3 |
| Min Speed = 20 | 0.81 | 0.93 | 330.1 |

Table S4 presents the performance of AI sonification as a function of the number of EEG channels utilized as input. Two-channel settings utilize only F4-C4 and F3-C3 EEG channels for sonification. The pairs of electrodes present in 8 and 18-channel montages can be found in Figure S5. The best performance is obtained with an 8-channel montage.

Table S4. Performance for the different number of EEG channels.

| #EEG channels | Sensitivity | Specificity |
| --- | --- | --- |
| 2 | 0.62 | 0.93 |
| **8** | **0.83** | **0.95** |
| 18 | 0.85 | 0.85 |

Figure S6 is an extension of Figure 5 in the main article that includes the results for the non-clinical interpreters who also participated in the survey. It shows the performance of both types of interpreters obtained from the AI sonification survey compared to the accuracy of the AI algorithm alone in terms of the AUC. The red/black mark indicates the performance obtained by the clinical (23) and non-clinical (12) interpreters, respectively. The clinical interpreters achieve higher sensitivity (0.89 vs 0.83) but lower specificity (0.78 vs 0.91) than that the non-clinical interpreters.

Figures Figure S7 and Figure S8 show additional demos of the method on seizure and background EEG recordings from the Helsinki dataset.


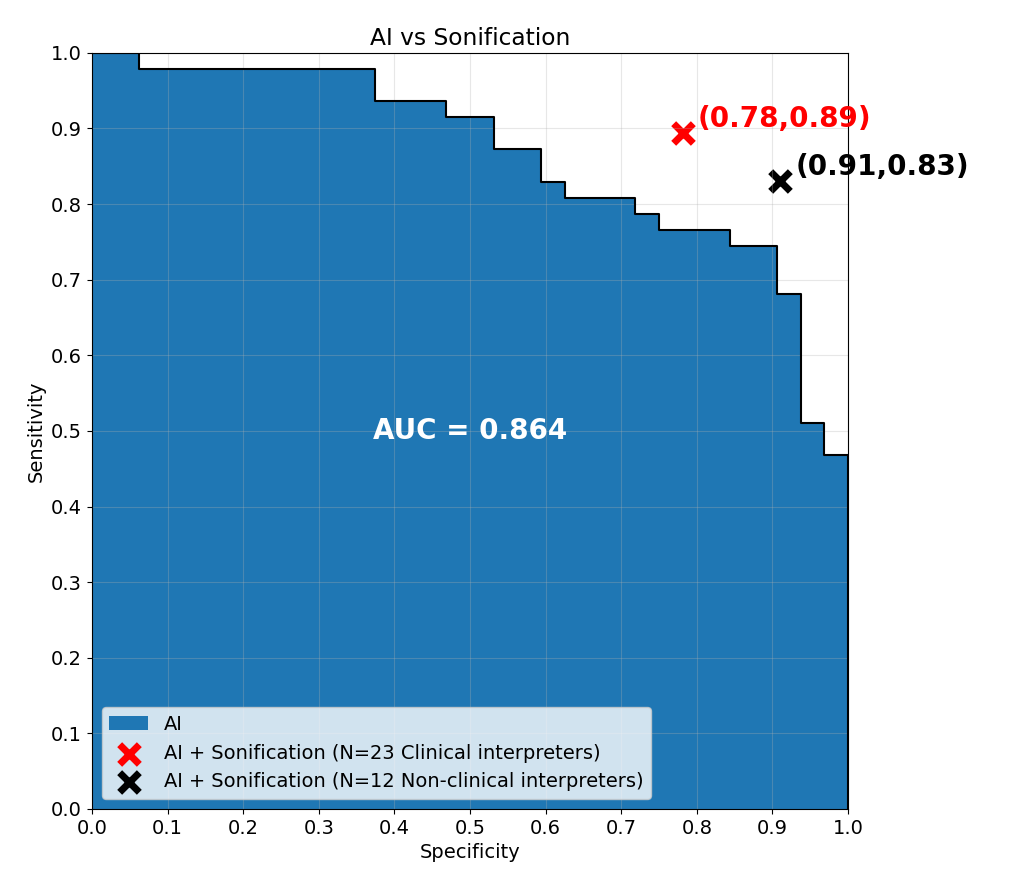


Figure S6. The area under the curve (AUC) for the AI probabilistic output and sensitivity/specificity of the majority vote of the survey participants, including clinical and non-clinical interpreters.


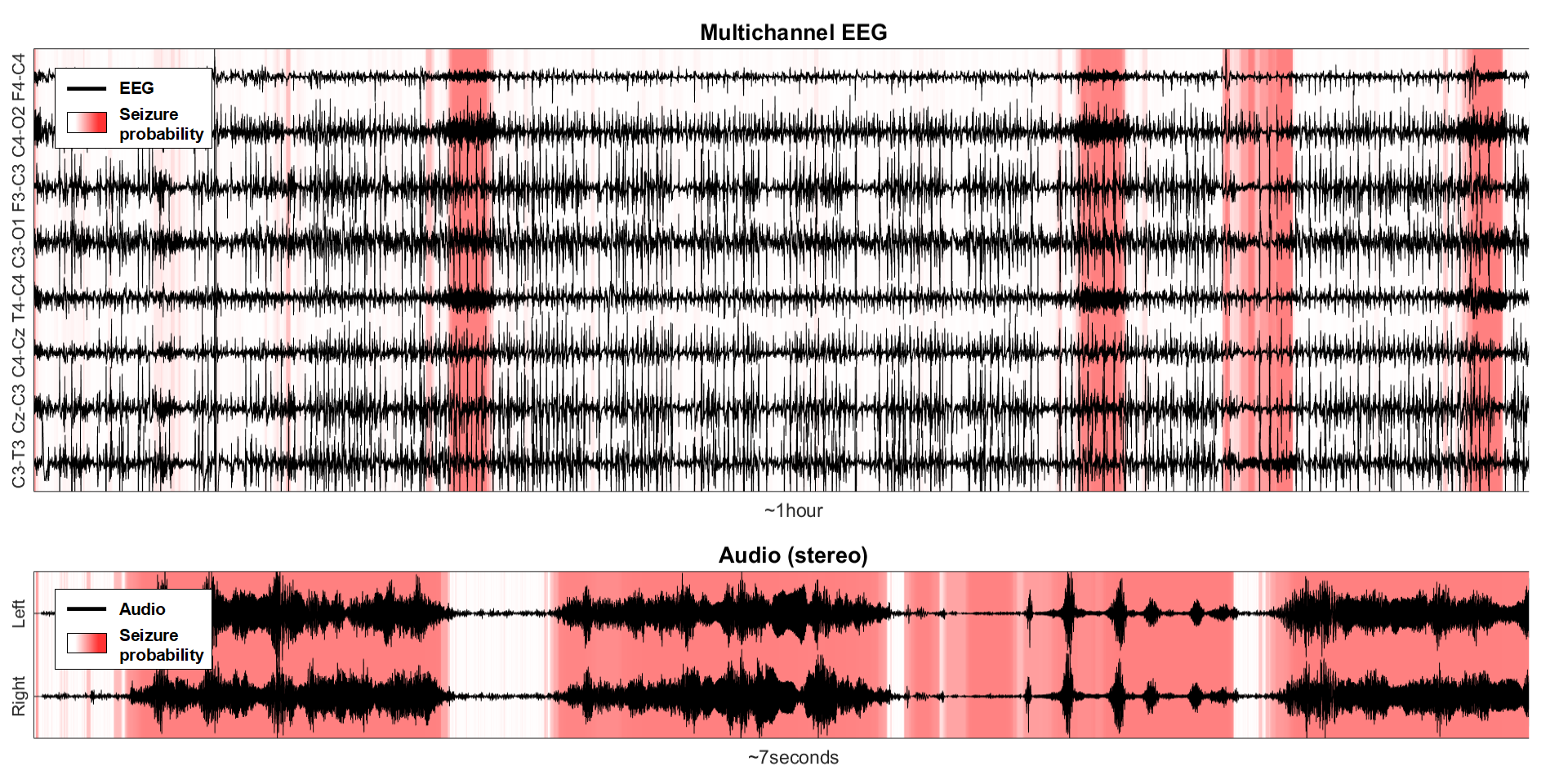


Figure S7. AI-driven sonification algorithm demo (EEG7 in Helsinki dataset). Supplementary example of EEG recording containing seizures (best seen in color).

The following are the sounds resulting from their respective EEG recordings in the Helsinki dataset:


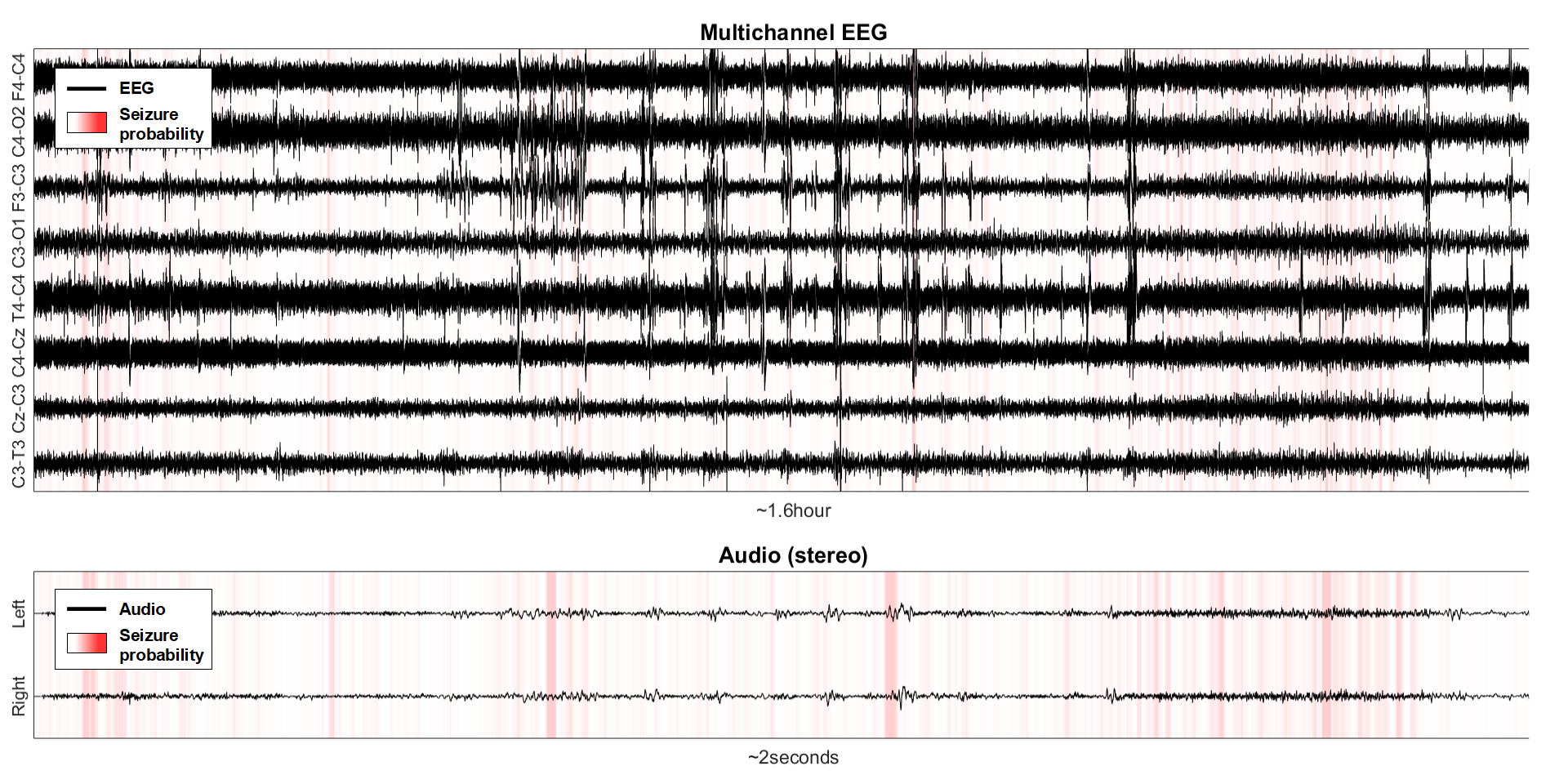


Figure S8. AI-driven sonification algorithm demo (EEG28 in Helsinki dataset). Supplementary example of EEG recording containing no seizures (best seen in color).

- [EEG31](https://sergigomezquintana.github.io/EEGsoundSurvey/html/audio/eeg31.mp3) (Figure 2 shown in the main manuscript).
- [EEG7](https://sergigomezquintana.github.io/EEGsoundSurvey/html/audio/eeg7.mp3) (Figure S7).
- [EEG28](https://sergigomezquintana.github.io/EEGsoundSurvey/html/audio/eeg28.mp3) (Figure S8).

# Supplementary Discussion

The Cohen kappa scores presented in Table S2 conveys a similar message than Figure 4 in the main document, but from a 1-to-1 perspective. Once again, this shows that the accuracy obtained through the interpretation of AI-assisted sonification of 1-2h segment of EEG is not statistically different from that of the experienced EEG interpreters who had access to full multi-channel EEG.

As with any new method, AI sonification requires several parameters to be tuned. Moreover, the tuning had to be done empirically since it is difficult to analytically assess the influence of internal settings on the resultant audio’s perceptual and discriminatory aspects. Table S3 condenses the explored parametric variations of the algorithm along with the results obtained from the internal survey.

It was found that while pitch evolution was indicative of seizures, the presence of constant pitch itself was perplexing to the listeners. The detection of evolving patterns was improved with more training examples. However, giving more examples to train comes at the cost of removing examples from the test. It also increases the overall survey duration. The main contributing factor to the constant pitch was found to be the presence of seizure-like artifacts, like sweating or ECG artifacts [15]. Incorporating the ECG artifact removal algorithm was proposed to attenuate the ECG interference. The resultant sound contained fewer seizure-like sounding segments.

Interestingly, the removal of ECG interference was expected to improve the specificity with fewer false positives. While the specificity was marginally improved, sensitivity’s largest gain went from 0.787 to 0.830. This confirms why it is difficult to analytically predict how perceptually the ear will respond to the changes in the algorithm.

Adjusting other parameters that control the duration of the resultant audio, such as the probability distribution or the Min Speed constant, did not result in an increase in Sensitivity or Specificity while resulting in a significant increase in the audio duration, with the compression factor decreasing from 727.4 to 359.3 for probability, and to 330.1 for Min Speed, showing that the method is relatively robust to parametric change. Conversely, in [16] aEEG was evaluated on non-expert, showing that there is a sensitivity/specificity trade-off as a function of the aEEG resolution (speed, mm/min), with 38/92% sens/spec at low-resolution (1 mm/min) and 55/61% at higher resolution (5 mm/min), demonstrating a stronger dependence on the parametric display setting of aEEG.

The public EEG dataset used in this study comes in the 18-channel montage. From the results from Table S4, it is clear that the usage of 8-channel montage gives clear performance advantages over the 2-channel montage for both Sensitivity and Specificity. The usage of 18-channel improves sensitivity from 0.830 to 0.851. An example was found (EEG sample #77) where the seizures are only audible in P3-O1 and C3-P3 channels, which are not included in the standard 8-channel montage. Likewise, it was shown that reducing the number of electrodes results in a significant reduction in seizure detection [17]. However, being exposed to 18 sources of information reduces specificity from 0.951 to 0.851. Thus, the performance gains were not conclusive to switch to the full 18-channel montage, and we kept the simpler 8-channel montage in the clinical survey.

One of the key features of the proposed method is that it allows for interpretability and subjectiveness across interpreters. From Figure S6, it can be seen that both clinical and non-clinical interpreters outperform the AI system. However, it is interesting to observe that clinicians achieve significantly higher sensitivity (0.89 vs 0.83) whereas the non-clinical interpreters have significantly higher specificity (0.78 vs 0.91). The higher sensitivity obtained by the clinical interpreters implies inherited bias of trained healthcare professionals to catch the disease. This bias affects the hearing so that even small pitch changes result in an alarm. This allows detection of seizure patients whom the non-clinical interpreters did not identify. In contrast, the non-clinical interpreters are much more conservative, only raising the alarm when confidently hearing something abnormal, with fewer false alarms than the clinical interpreters.

The twelve non-clinical interpreters were mainly designed to indicate the performance obtained with lay users. The non-clinical interpreter may not fully understand the underlying physiological processes for diagnosing seizures or the medical consequences of such decisions. The invitation of the non-clinical interpreter to participate in the survey was mostly driven by curiosity regarding the level of the performance and the location of the operating point. Twelve participants were sufficient to show a similar level of performance and the existence of a different operating point on the curve of AI-driven sonification.

Figures Figure S7 and Figure S8 provide additional illustrations on seizure and no-seizure examples (respectively) on how the proposed method works. Since AI is used as an attention mechanism to modulate the playback speed of the resulting audio, a 1-hour segment of EEG data that contains seizures is converted into a 7s of audio, while a longer 1.6-hour segment with no seizures is conversely converted into just 2s of audio. Moreover, the waveform amplitude on the resulting audio from the example containing seizures (Figure S7) is much larger compared to the seizure-free example (Figure S8), which results in a more silent sound.

# References

| [1] | White, D. M. & Van Cott, A. C. EEG artifacts in the intensive care unit setting. *Neurodiagnostic Journal* vol. 50 8–25 (2010) |
| --- | --- |
| [2] | Boll, S. F. Suppression of Acoustic Noise in Speech Using Spectral Subtraction. *IEEE Transactions on Acoustics, Speech, and Signal Processing* **27**, 113–120 (1979) |
| [3] | Flanagan, J. L. & Golden, R. M. Phase Vocoder. *Bell System Technical Journal* **45**, 1493–1509 (1966) |
| [4] | Laroche, J. & Dolson, M. Improved phase vocoder time-scale modification of audio. *IEEE Transactions on Speech and Audio Processing* **7**, 323–332 (1999) |
| [5] | Temko, A. Estimation of heart rate from photoplethysmography during physical exercise using Wiener filtering and the phase vocoder. in *Proceedings of the Annual International Conference of the IEEE Engineering in Medicine and Biology Society, EMBS* vols 2015-Novem 1500–1503 (2015). |
| [6] | McGee, R. & Rogers, D. Musification of Seismic Data. in *International Conference on Auditory Display* 201–204 (2017). |
| [7] | Dolson, M. Phase Vocoder: A tutorial. *Computer Music Journal* **10**, 14–27 (1986) |
| [8] | O’Shea, A., Lightbody, G., Boylan, G. & Temko, A. Neonatal seizure detection from raw multi-channel EEG using a fully convolutional architecture. *Neural Networks* **123**, 12–25 (2020) |
| [9] | Temko, A., Marnane, W., Boylan, G., O’Toole, J. M. & Lightbody, G. Neonatal EEG audification for seizure detection. in *2014 36th Annual International Conference of the IEEE Engineering in Medicine and Biology Society, EMBC 2014* 4451–4454 (2014). |
| [10] | Brown, C. P. & Duda, R. O. Efficient HRTF model for 3-D sound. in *IEEE ASSP Workshop on Applications of Signal Processing to Audio and Acoustics* (1997). |
| [11] | Algazi, V. R., Duda, R. O., Duraiswami, R., Gumerov, N. A. & Tang, Z. Approximating the head-related transfer function using simple geometric models of the head and torso. *The Journal of the Acoustical Society of America* **112**, 2053–2064 (2002) |
| [12] | Kapralos, B., Jenkin, M. R. & Milios, E. Virtual audio systems. *Presence: Teleoperators and Virtual Environments* **17**, 527–549 (2008) |
| [13] | Middlebrooks, J. C. Sound localization. in *Handbook of Clinical Neurology* vol. 129 99–116 (2015). |
| [14] | Brown, A. D., Stecker, G. C. & Tollin, D. J. The Precedence Effect in Sound Localization. *Journal of the Association for Research in Otolaryngology 2014 16:1* **16**, 1–28 (2014) |
| [15] | White, D. M. & Van Cott, A. C. EEG artifacts in the intensive care unit setting. *Neurodiagnostic Journal* vol. 50 8–25 (2010) |
| [16] | Rakshasbhuvankar, A., Paul, S., Nagarajan, L., Ghosh, S. & Rao, S. Amplitude-integrated EEG for detection of neonatal seizures: a systematic review. *Seizure* **33**, 90–98 (2015) |
| [17] | Stevenson, N., Lauronen, L. & Vanhatalo, S. The effect of reducing EEG electrode number on the visual interpretation of the human expert for neonatal seizure detection. *Clinical Neurophysiology* **129**, 265–270 (2018) |

# Figure Legends

Figure S1. From top to bottom: Recorded ECG signal, recorded EEG (single channel) and cleaned EEG with ECG artifact removed.

Figure S2. General scheme for input STFT X[n,k] and phase vocoder output STFT Y[m,k].

Figure S3. VSPV examples. (a) Tones of 8, 16 and 32Hz stretched/contracted individually. (b) Chirp signal being stretched continuously.

Figure S4. Precedence effect due to interaural delay.

Figure S5. Approximate locations on the scalp of the 8 and 18 channel montages for EEG acquisition.

Figure S6. The area under the curve (AUC) for the AI probabilistic output and sensitivity/specificity of the majority vote of the survey participants, including clinical and non-clinical interpreters.

Figure S7. AI-driven sonification algorithm demo (EEG7 in Helsinki dataset). Supplementary example of EEG recording containing seizures (best seen in color).

Figure S8. AI-driven sonification algorithm demo (EEG28 in Helsinki dataset). Supplementary example of EEG recording containing no seizures (best seen in color).

# Table Legends

Table S1. Polar coordinates of electrodes. (left-hemisphere only).

Table S2. Cohen’s kappa scores for annotator pairs and AI sonification algorithm (Kappa ± CI95).

Table S3. The influence of the parameters on the performance of AI sonification, in terms of sensitivity, specificity and compression rate.

Table S4. Performance for the different number of EEG channels.
